# Functional mechanisms underlying pleiotropic risk alleles at the 19p13.1 breast–ovarian cancer susceptibility locus

**DOI:** 10.1038/ncomms12675

**Published:** 2016-09-07

**Authors:** Kate Lawrenson, Siddhartha Kar, Karen McCue, Karoline Kuchenbaeker, Kyriaki Michailidou, Jonathan Tyrer, Jonathan Beesley, Susan J. Ramus, Qiyuan Li, Melissa K. Delgado, Janet M. Lee, Kristiina Aittomäki, Irene L. Andrulis, Hoda Anton-Culver, Volker Arndt, Banu K. Arun, Brita Arver, Elisa V. Bandera, Monica Barile, Rosa B. Barkardottir, Daniel Barrowdale, Matthias W. Beckmann, Javier Benitez, Andrew Berchuck, Maria Bisogna, Line Bjorge, Carl Blomqvist, William Blot, Natalia Bogdanova, Anders Bojesen, Stig E. Bojesen, Manjeet K. Bolla, Bernardo Bonanni, Anne-Lise Børresen-Dale, Hiltrud Brauch, Paul Brennan, Hermann Brenner, Fiona Bruinsma, Joan Brunet, Shaik Ahmad Buhari, Barbara Burwinkel, Ralf Butzow, Saundra S. Buys, Qiuyin Cai, Trinidad Caldes, Ian Campbell, Rikki Cannioto, Jenny Chang-Claude, Jocelyne Chiquette, Ji-Yeob Choi, Kathleen B. M. Claes, GEMO Study Collaborators, Marie- Agnès Collonge-Rame, Alexandre Damette, Emmanuelle Barouk-Simonet, Françoise Bonnet, Virginie Bubien, Nicolas Sevenet, Michel Longy, Pascaline Berthet, Dominique Vaur, Laurent Castera, Sandra Fert Ferrer, Yves-Jean Bignon, Nancy Uhrhammer, Fanny Coron, Laurence Faivre, Amandine Baurand, Caroline Jacquot, Geoffrey Bertolone, Sarab Lizard, Dominique Leroux, Hélène Dreyfus, Christine Rebischung, Magalie Peysselon, Jean-Philippe Peyrat, Joëlle Fournier, Françoise Révillion, Claude Adenis, Laurence Vénat-Bouvet, Mélanie Léone, Nadia Boutry-Kryza, Alain Calender, Sophie Giraud, Carole Verny-Pierre, Christine Lasset, Valérie Bonadona, Laure Barjhoux, Hagay Sobol, Violaine Bourdon, Tetsuro Noguchi, Audrey Remenieras, Isabelle Coupier, Pascal Pujol, Johanna Sokolowska, Myriam Bronner, Capucine Delnatte, Stéphane Bézieau, Véronique Mari, Marion Gauthier-Villars, Bruno Buecher, Etienne Rouleau, Lisa Golmard, Virginie Moncoutier, Muriel Belotti, Antoine de Pauw, Camille Elan, Emmanuelle Fourme, Anne-Marie Birot, Claire Saule, Maïté Laurent, Claude Houdayer, Fabienne Lesueur, Noura Mebirouk, Florence Coulet, Chrystelle Colas, Florent Soubrier, Mathilde Warcoin, Fabienne Prieur, Marine Lebrun, Caroline Kientz, Danièle Muller, Jean-Pierre Fricker, Christine Toulas, Rosine Guimbaud, Laurence Gladieff, Viviane Feillel, Isabelle Mortemousque, Brigitte Bressac-de-Paillerets, Olivier Caron, Marine Guillaud-Bataille, Linda S. Cook, Angela Cox, Daniel W. Cramer, Simon S. Cross, Cezary Cybulski, Kamila Czene, Mary B. Daly, Francesca Damiola, Agnieszka Dansonka-Mieszkowska, Hatef Darabi, Joe Dennis, Peter Devilee, Orland Diez, Jennifer A. Doherty, Susan M. Domchek, Cecilia M. Dorfling, Thilo Dörk, Martine Dumont, Hans Ehrencrona, Bent Ejlertsen, Steve Ellis, EMBRACE, Helen Gregory, Zosia Miedzybrodzka, Patrick J. Morrison, Alan Donaldson, Mark T. Rogers, M. John Kennedy, Mary E. Porteous, Angela Brady, Julian Barwell, Claire Foo, Fiona Lalloo, Lucy E. Side, Jacqueline Eason, Alex Henderson, Lisa Walker, Jackie Cook, Katie Snape, Alex Murray, Emma McCann, Christoph Engel, Eunjung Lee, D. Gareth Evans, Peter A. Fasching, Lidia Feliubadalo, Jonine Figueroa, Dieter Flesch-Janys, Olivia Fletcher, Henrik Flyger, Lenka Foretova, Florentia Fostira, William D. Foulkes, Brooke L. Fridley, Eitan Friedman, Debra Frost, Gaetana Gambino, Patricia A. Ganz, Judy Garber, Montserrat García-Closas, Aleksandra Gentry-Maharaj, Maya Ghoussaini, Graham G. Giles, Rosalind Glasspool, Andrew K. Godwin, Mark S. Goldberg, David E. Goldgar, Anna González-Neira, Ellen L. Goode, Marc T. Goodman, Mark H. Greene, Jacek Gronwald, Pascal Guénel, Christopher A. Haiman, Per Hall, Emily Hallberg, Ute Hamann, Thomas V. O. Hansen, Patricia A. Harrington, Mikael Hartman, Norhashimah Hassan, Sue Healey, The Hereditary Breast and Ovarian Cancer Research Group Netherlands (HEBON), M. A. Rookus, F. E. van Leeuwen, L. E. van der Kolk, M. K. Schmidt, N. S. Russell, J. L. de Lange, R. Wijnands, J. M. Collée, M. J. Hooning, C. Seynaeve, C. H. M. van Deurzen, I. M. Obdeijn, C. J. van Asperen, R. A. E. M. Tollenaar, T. C. T. E. F. van Cronenburg, C. M. Kets, M. G. E. M. Ausems, C. C. van der Pol, T. A. M. van Os, Q. Waisfisz, H. E. J. Meijers-Heijboer, E. B. Gómez-Garcia, J. C. Oosterwijk, M. J. Mourits, G. H. de Bock, H. F. Vasen, S. Siesling, J. Verloop, L. I. H. Overbeek, Florian Heitz, Josef Herzog, Estrid Høgdall, Claus K. Høgdall, Frans B. L. Hogervorst, Antoinette Hollestelle, John L. Hopper, Peter J. Hulick, Tomasz Huzarski, Evgeny N. Imyanitov, KConFab Investigators, Stephen Fox, Judy Kirk, Geoff Lindeman, Melanie Price, Australian Ovarian Cancer Study Group, David Bowtell, Anna deFazio, Penny Webb, Claudine Isaacs, Hidemi Ito, Anna Jakubowska, Ramunas Janavicius, Allan Jensen, Esther M. John, Nichola Johnson, Maria Kabisch, Daehee Kang, Miroslav Kapuscinski, Beth Y. Karlan, Sofia Khan, Lambertus A. Kiemeney, Susanne Kruger Kjaer, Julia A. Knight, Irene Konstantopoulou, Veli-Matti Kosma, Vessela Kristensen, Jolanta Kupryjanczyk, Ava Kwong, Miguel de la Hoya, Yael Laitman, Diether Lambrechts, Nhu Le, Kim De Leeneer, Jenny Lester, Douglas A. Levine, Jingmei Li, Annika Lindblom, Jirong Long, Artitaya Lophatananon, Jennifer T. Loud, Karen Lu, Jan Lubinski, Arto Mannermaa, Siranoush Manoukian, Loic Le Marchand, Sara Margolin, Frederik Marme, Leon F. A. G. Massuger, Keitaro Matsuo, Sylvie Mazoyer, Lesley McGuffog, Catriona McLean, Iain McNeish, Alfons Meindl, Usha Menon, Arjen R. Mensenkamp, Roger L. Milne, Marco Montagna, Kirsten B. Moysich, Kenneth Muir, Anna Marie Mulligan, Katherine L. Nathanson, Roberta B. Ness, Susan L. Neuhausen, Heli Nevanlinna, Silje Nord, Robert L. Nussbaum, Kunle Odunsi, Kenneth Offit, Edith Olah, Olufunmilayo I. Olopade, Janet E. Olson, Curtis Olswold, David O’Malley, Irene Orlow, Nick Orr, Ana Osorio, Sue Kyung Park, Celeste L. Pearce, Tanja Pejovic, Paolo Peterlongo, Georg Pfeiler, Catherine M. Phelan, Elizabeth M. Poole, Katri Pylkäs, Paolo Radice, Johanna Rantala, Muhammad Usman Rashid, Gad Rennert, Valerie Rhenius, Kerstin Rhiem, Harvey A. Risch, Gus Rodriguez, Mary Anne Rossing, Anja Rudolph, Helga B. Salvesen, Suleeporn Sangrajrang, Elinor J. Sawyer, Joellen M. Schildkraut, Marjanka K. Schmidt, Rita K. Schmutzler, Thomas A. Sellers, Caroline Seynaeve, Mitul Shah, Chen-Yang Shen, Xiao-Ou Shu, Weiva Sieh, Christian F. Singer, Olga M. Sinilnikova, Susan Slager, Honglin Song, Penny Soucy, Melissa C. Southey, Marie Stenmark-Askmalm, Dominique Stoppa-Lyonnet, Christian Sutter, Anthony Swerdlow, Sandrine Tchatchou, Manuel R. Teixeira, Soo H. Teo, Kathryn L. Terry, Mary Beth Terry, Mads Thomassen, Maria Grazia Tibiletti, Laima Tihomirova, Silvia Tognazzo, Amanda Ewart Toland, Ian Tomlinson, Diana Torres, Thérèse Truong, Chiu-chen Tseng, Nadine Tung, Shelley S. Tworoger, Celine Vachon, Ans M. W. van den Ouweland, Helena C. van Doorn, Elizabeth J. van Rensburg, Laura J. Van't Veer, Adriaan Vanderstichele, Ignace Vergote, Joseph Vijai, Qin Wang, Shan Wang-Gohrke, Jeffrey N. Weitzel, Nicolas Wentzensen, Alice S. Whittemore, Hans Wildiers, Robert Winqvist, Anna H. Wu, Drakoulis Yannoukakos, Sook-Yee Yoon, Jyh-Cherng Yu, Wei Zheng, Ying Zheng, Kum Kum Khanna, Jacques Simard, Alvaro N. Monteiro, Juliet D. French, Fergus J. Couch, Matthew L. Freedman, Douglas F. Easton, Alison M. Dunning, Paul D. Pharoah, Stacey L. Edwards, Georgia Chenevix-Trench, Antonis C. Antoniou, Simon A. Gayther

**Affiliations:** 1 Department of Preventive Medicine, Keck School of Medicine, University of Southern California Norris Comprehensive Cancer Center, Los Angeles, 90033 California USA; 2 Department of Oncology, Centre for Cancer Genetic Epidemiology, University of Cambridge, Cambridge, CB1 8RN UK; 3 QIMR Berghofer Medical Research Institute, Brisbane, 4029 Queensland Australia; 4 Department of Public Health and Primary Care, Centre for Cancer Genetic Epidemiology, University of Cambridge, Cambridge, CB1 8RN UK; 5 Medical College, Xiamen University, Xiamen, 361102 China; 6 Department of Medical Oncology, The Center for Functional Cancer Epigenetics, Dana-Farber Cancer Institute, Boston, 02215 Massachusetts USA; 7 Department of Clinical Genetics, Helsinki University Hospital, University of Helsinki, Helsinki, 00029 HUS Finland; 8 Lunenfeld-Tanenbaum Research Institute of Mount Sinai Hospital, Toronto, M5G 1X5 Ontario Canada; 9 Department of Molecular Genetics, University of Toronto, Toronto, M5S 1A8 Ontario Canada; 10 Department of Epidemiology, Genetic Epidemiology Research Institute, School of Medicine, University of California Irvine, Irvine, 92697 California USA; 11 Division of Clinical Epidemiology and Aging Research, German Cancer Research Center (DKFZ), Heidelberg, 69120 Germany; 12 University of Texas MD Anderson Cancer Center, Houston, 77030 Texas USA; 13 Department of Oncology, Karolinska University Hospital, Stockholm, 171 77 Sweden; 14 Cancer Prevention and Control, Rutgers Cancer Institute of New Jersey, New Brunswick, 08903 New Jersey USA; 15 Division of Cancer Prevention and Genetics, Istituto Europeo di Oncologia, Milan, 20141 Italy; 16 Department of Pathology, Landspitali University Hospital and BMC (Biomedical Centre), Faculty of Medicine, University of Iceland, Reykjavik, 600169-2039 Iceland; 17 Department of Gynecology and Obstetrics, University Hospital Erlangen, Friedrich-Alexander-University Erlangen-Nuremberg, Comprehensive Cancer Center Erlangen-EMN, Erlangen, 91054 Germany; 18 Human Cancer Genetics Program, Spanish National Cancer Research Centre, Madrid, E-28029 Spain; 19 Centro de Investigación en Red de Enfermedades Raras, Valencia, 28029 Spain; 20 Department of Obstetrics and Gynecology, Duke University Medical Center, Durham, 27710 North Carolina USA; 21 Department of Surgery, Gynecology Service, Memorial Sloan-Kettering Cancer Center, New York, 10065 USA; 22 Department of Gynecology and Obstetrics, Haukeland University Hospital, Bergen, 5021 Norway; 23 Department of Clinical Science, Centre for Cancer Biomarkers, University of Bergen, Bergen, N-5020 Norway; 24 Department of Oncology, Helsinki University Hospital, University of Helsinki, Helsinki, FIN-00029 Finland; 25 Division of Epidemiology, Department of Medicine, Vanderbilt-Ingram Cancer Center, Vanderbilt University School of Medicine, Nashville, 37203 Tennessee USA; 26 International Epidemiology Institute, Rockville, 20850 Maryland USA; 27 Gynaecology Research Unit, Hannover Medical School, Hannover, D-30625 Germany; 28 Department of Clinical Genetics, Vejle Hospital, Vejle, 7100 Denmark; 29 Faculty of Health and Medical Sciences, University of Copenhagen, Copenhagen, 2200 Denmark; 30 Department of Clinical Biochemistry, Herlev Hospital, Copenhagen University Hospital, Herlev, 2730 Denmark; 31 Copenhagen General Population Study, Herlev Hospital, Copenhagen University Hospital, Herlev, 2730 Denmark; 32 Department of Genetics, Institute for Cancer Research, Oslo University Hospital Radiumhospitalet, Oslo, N-0310 Norway; 33 K.G. Jebsen Center for Breast Cancer Research, Institute of Clinical Medicine, Faculty of Medicine, University of Oslo, Oslo, N-0310 Norway; 34 Dr Margarete Fischer-Bosch-Institute of Clinical Pharmacology, Stuttgart, D-70376 Germany; 35 University of Tübingen, Tübingen, 72074 Germany; 36 German Cancer Consortium (DKTK), German Cancer Research Center (DKFZ), Heidelberg, 69120 Germany; 37 International Agency for Research on Cancer, Lyon, 69008 France; 38 Division of Preventive Oncology, German Cancer Research Center (DKFZ), Heidelberg, 69121 Germany; 39 Cancer Epidemiology Centre, Cancer Council Victoria, Melbourne, 3004 Victoria Australia; 40 Genetic Counseling Unit, Hereditary Cancer Program, IDIBGI (Institut d'Investigació Biomèdica de Girona), Catalan Institute of Oncology, Girona, 08908 Spain; 41 Department of Surgery, National University Health System, Singapore, 119077 Singapore; 42 Molecular Epidemiology Group, German Cancer Research Center (DKFZ), Heidelberg, 69120 Germany; 43 Department of Obstetrics and Gynecology, University of Heidelberg, Heidelberg, 69120 Germany; 44 Department of Obstetrics and Gynecology, University of Helsinki and Helsinki University Central Hospital, Helsinki, 00029 HUS Finland; 45 Department of Pathology, Helsinki University Central Hospital, Helsinki, 00029 Finland; 46 Department of Medicine, Huntsman Cancer Institute, University of Utah School of Medicine, Salt Lake City, 84112 Utah USA; 47 Molecular Oncology Laboratory, Hospital Clinico San Carlos, IdISSC (El Instituto de Investigación Sanitaria del Hospital Clínico San Carlos), Madrid, 28040 Spain; 48 Cancer Genetics Laboratory, Peter MacCallum Cancer Centre, Melbourne, 3002 Victoria Australia; 49 Division of Cancer Prevention and Population Sciences, Cancer Pathology & Prevention, Roswell Park Cancer Institute, Elm and Carlton Streets, Buffalo, 14263 New York USA; 50 Division of Cancer Epidemiology, German Cancer Research Center (DKFZ), Heidelberg, 69121 Germany; 51 University Cancer Center Hamburg (UCCH), University Medical Center Hamburg-Eppendorf, Hamburg, 20246 Germany; 52 Unité de recherche en santé des populations, Centre des maladies du sein Deschênes-Fabia, Centre de recherche FRSQ du Centre hospitalier affilié universitaire de Québec, Québec City, G1J 1Z4 Québec Canada; 53 Cancer Research Institute, Seoul National University, Seoul, 08826 Korea; 54 Department of Biomedical Sciences, Seoul National University College of Medicine, Seoul, 03080 Korea; 55 Center for Medical Genetics, Ghent University, Ghent, 9000 Belgium; 56 Division of Epidemiology and Biostatistics, Department of Internal Medicine, University of New Mexico, Albuquerque, 87131 New Mexico USA; 57 Department of Oncology, Sheffield Cancer Research, University of Sheffield, Sheffield, S10 2TN UK; 58 Harvard HT Chan School of Public Health, Boston, 02115 Massachusetts USA; 59 Obstetrics and Gynecology Epidemiology Center, Brigham and Women's Hospital and Harvard Medical School, Boston, 02115 Massachusetts USA; 60 Department of Neuroscience, Academic Unit of Pathology, University of Sheffield, Sheffield, S10 2TN UK; 61 Department of Genetics and Pathology, Pomeranian Medical University, Szczecin, 70-115 Poland; 62 Department of Medical Epidemiology and Biostatistics, Karolinska Institutet, Stockholm, SE-171 77 Sweden; 63 Department of Clinical Genetics, Fox Chase Cancer Center, Philadelphia, 19111 Pennsylvania USA; 64 INSERM U1052, CNRS UMR5286, Université Lyon, Centre de Recherche en Cancérologie de Lyon, Lyon, 69373 France; 65 Department of Pathology and Laboratory Diagnostics the Maria Sklodowska Curie Memorial Cancer Center and Institute of Oncology, Warsaw, 44-101 Poland; 66 Department of Pathology, Leiden University Medical Center, Leiden, 2333 The Netherlands; 67 Department of Human Genetics, Leiden University Medical Center, Leiden, 2333 The Netherlands; 68 Oncogenetics Group, University Hospital Vall d’Hebron, Vall d’Hebron Institute of Oncology (VHIO) and Universitat Autònoma de Barcelona, Barcelona, 08035 Spain; 69 Department of Community and Family Medicine, Section of Biostatistics & Epidemiology, The Geisel School of Medicine at Dartmouth, Lebanon, 03755 New Hampshire USA; 70 Department of Medicine, Abramson Cancer Center, Perelman School of Medicine, University of Pennsylvania, Philadelphia, 19104 Pennsylvania USA; 71 Department of Genetics, University of Pretoria, Pretoria, 0083 South Africa; 72 Genomics Center, Centre Hospitalier Universitaire de Québec Research Center, Laval University, Québec City, G1V 4G2 Québec Canada; 73 Department of Immunology, Genetics and Pathology, Uppsala University, Uppsala, 751 05 Sweden; 74 Department of Clinical Genetics, Lund University Hospital, Lund, 221 00 Sweden; 75 Department of Oncology, Rigshospitalet, Copenhagen University Hospital, Copenhagen, 2100 Denmark; 76 Institute for Medical Informatics, Statistics and Epidemiology, University of Leipzig, Leipzig, 04107 Germany; 77 Genomic Medicine, Manchester Academic Health Sciences Centre, Institute of Human Development, Manchester University, Central Manchester University Hospitals NHS Foundation Trust, Manchester, M13 9PL UK; 78 Department of Medicine, Division of Hematology and Oncology, University of California at Los Angeles, David Geffen School of Medicine, Los Angeles, 90095 California USA; 79 Molecular Diagnostic Unit, Hereditary Cancer Program, IDIBELL (Bellvitge Biomedical Research Institute),Catalan Institute of Oncology, Barcelona, 08908 Spain; 80 Division of Cancer Epidemiology and Genetics, National Cancer Institute, Rockville, 20892 Maryland USA; 81 Institute for Medical Biometrics and Epidemiology, University Medical Center Hamburg-Eppendorf, Hamburg, 20246 Germany; 82 Department of Cancer Epidemiology, Clinical Cancer Registry, University Medical Center Hamburg-Eppendorf, Hamburg, 20246 Germany; 83 Breakthrough Breast Cancer Research Centre, The Institute of Cancer Research, London, SW3 6JB UK; 84 Division of Breast Cancer Research, The Institute of Cancer Research, London, SW7 3RP UK; 85 Department of Breast Surgery, Herlev Hospital, Copenhagen University Hospital, Herlev, 2730 Denmark; 86 Masaryk Memorial Cancer Institute and Medical Faculty MU, Brno, 625 00 Czech Republic; 87 Molecular Diagnostics Laboratory, (INRASTES) Institute of Nuclear and Radiological Sciences and Technology, National Centre for Scientific Research ‘Demokritos’, Aghia Paraskevi Attikis, Athens, 153 10 Greece; 88 Departments of Human Genetics and Oncology, Program in Cancer Genetics, McGill University, Montreal, Montreal, H2W 1S6 Quebec Canada; 89 Biostatistics and Informatics Shared Resource, University of Kansas Medical Center, Kansas City, 66160 Kansas USA; 90 Susanne Levy Gertner Oncogenetics Unit, Sheba Medical Center, Tel-Hashomer, 52621 Israel; 91 Department of Laboratory Medicine, Section of Genetic Oncology, University and University Hospital of Pisa, Pisa, 56126 Italy; 92 Division of Cancer Prevention & Control Research, UCLA Schools of Medicine and Public Health, Jonsson Comprehensive Cancer Center, Los Angeles, 90024 California USA; 93 Cancer Risk and Prevention Clinic, Dana-Farber Cancer Institute, Boston, 02215 Massachusetts USA; 94 Division of Genetics and Epidemiology, The Institute of Cancer Research, London, SW7 3RP UK; 95 Women's Cancer, UCL EGA Institute for Women's Health, London, WC1E 6AU UK; 96 Centre for Epidemiology and Biostatistics, Melbourne School of Population and Global Health, The University of Melbourne, Melbourne, 3010 Victoria Australia; 97 Cancer Research UK Clinical Trials Unit, The Beatson West of Scotland Cancer Centre, Glasgow, G12 0YN UK; 98 Department of Pathology and Laboratory Medicine, University of Kansas Medical Center, Kansas City, 66160 Kansas USA; 99 Division of Clinical Epidemiology, Royal Victoria Hospital, McGill University, Montreal, H3A 1A1 Québec Canada; 100 Department of Medicine, McGill University, Montreal, H3A 1A1 Québec Canada; 101 Department of Dermatology, Huntsman Cancer Institute, University of Utah School of Medicine, Salt Lake City, 84132 Utah USA; 102 Department of Health Sciences Research, Mayo Clinic, Rochester, 55902 Minnesota USA; 103 Cancer Prevention and Control, Samuel Oschin Comprehensive Cancer Institute, Cedars-Sinai Medical Center, Los Angeles, 90048 California USA; 104 Department of Biomedical Sciences, Community and Population Health Research Institute, Cedars-Sinai Medical Center, Los Angeles, 90048 California USA; 105 Division of Cancer Epidemiology and Genetics, Clinical Genetics Branch, National Cancer Institute, National Institutes of Health, Rockville, 20892 Maryland USA; 106 Department of Genetics and Pathology, Pomeranian Medical University, Szczecin, 70-204 Poland; 107 Environmental Epidemiology of Cancer, Center for Research in Epidemiology and Population Health, INSERM, Villejuif, 94805 France; 108 University Paris-Sud, Villejuif, 91405 France; 109 Molecular Genetics of Breast Cancer, German Cancer Research Center (DKFZ), Heidelberg, 69120 Germany; 110 Center for Genomic Medicine, Rigshospitalet, Copenhagen University Hospital, Copenhagen, 2100 Denmark; 111 Department of Oncology, Department of Public Health and Primary Care, University of Cambridge, Strangeways Research Laboratory, Cambridge, CB1 8RN UK; 112 Saw Swee Hock School of Public Health, National University of Singapore, Singapore, 119077 Singapore; 113 Breast Cancer Research Unit, Cancer Research Institute, University Malaya Medical Centre, Kuala Lumpur, 50603 Malaysia; 114 Cancer Research Initiatives Foundation, Subang Jaya, Selangor, 47500 Malaysia; 115 Department of Gynecology and Gynecologic Oncology, Kliniken Essen-Mitte, Essen, 45136 Germany; 116 Department of Gynecology and Gynecologic Oncology, Dr Horst Schmidt Kliniken Wiesbaden, Wiesbaden, 65199 Germany; 117 Clinical Cancer Genetics, for the City of Hope Clinical Cancer Genetics Community Research Network, Duarte, 91010 California USA; 118 Department of Pathology, Molecular Unit, Herlev Hospital, University of Copenhagen, Copenhagen, 2730 Denmark; 119 Department of Virus, Lifestyle and Genes, Danish Cancer Society Research Center, Copenhagen, DK-2100 Denmark; 120 Department of Gynecology, Rigshospitalet, University of Copenhagen, Copenhagen, 2100 Denmark; 121 Family Cancer Clinic, Netherlands Cancer Institute, Amsterdam, 1006 The Netherlands; 122 Department of Medical Oncology, Family Cancer Clinic, Erasmus MC Cancer Institute, Rotterdam, 3015 The Netherlands; 123 Center for Medical Genetics, NorthShore University Health System, Evanston, 60201 Illinois USA; 124 N.N. Petrov Institute of Oncology, St Petersburg, 197758 Russia; 125 Lombardi Comprehensive Cancer Center, Georgetown University, Washington, 20057 District of Columbia USA; 126 Division of Epidemiology and Prevention, Aichi Cancer Center Research Institute, Aichi, 464-8681 Japan; 127 State Research Institute Centre for Innovative Medicine, Vilnius, LT-01102 Lithuania; 128 Department of Epidemiology, Cancer Prevention Institute of California, Fremont, 94538 California USA; 129 Department of Preventive Medicine, Seoul National University College of Medicine, Seoul, 08826 Korea; 130 Centre for Epidemiology and Biostatistics, University of Melbourne, Melbourne, 3010 Victoria Australia; 131 Women's Cancer Program at the Samuel Oschin Comprehensive Cancer Institute, Cedars-Sinai Medical Center, Los Angeles, 90048 California USA; 132 Radboud University Medical Centre, Radboud Institute for Health Sciences, Nijmegen, 6500 The Netherlands; 133 Prosserman Centre for Health Research, Lunenfeld-Tanenbaum Research Institute of Mount Sinai Hospital, Toronto, M5G 1X5 Ontario Canada; 134 Division of Epidemiology, Dalla Lana School of Public Health, University of Toronto, Toronto, M5T 3M7 Ontario Canada; 135 Department of Clinical Pathology, Imaging Center, Kuopio University Hospital, Kuopio, 70210 Finland; 136 Cancer Center, Kuopio University Hospital, Kuopio, 70210 Finland; 137 Institute of Clinical Medicine, Pathology and Forensic Medicine, University of Eastern Finland, Kuopio, 70210 Finland; 138 Department of Clinical Molecular Biology, Oslo University Hospital, University of Oslo, 1478 Oslo Norway; 139 The Hong Kong Hereditary Breast Cancer Family Registry, Cancer Genetics Center, Hong Kong Sanatorium and Hospital, Hong Kong, China; 140 Department of Surgery, The University of Hong Kong, Hong Kong, China; 141 Vesalius Research Center, VIB, Leuven, 3000 Belgium; 142 Department of Oncology, Laboratory for Translational Genetics, University of Leuven, Leuven, 3000 Belgium; 143 Department of Molecular Medicine and Surgery, Karolinska Institutet, Stockholm, SE-171 77 Sweden; 144 Division of Health Sciences, Warwick Medical School, Warwick University, Coventry, CV4 7AL UK; 145 Department of Gynecologic Oncology, The University of Texas MD Anderson Cancer Center, Houston, 77030 Texas USA; 146 Department of Preventive and Predictive Medicine, Unit of Medical Genetics, Fondazione IRCCS (Istituto Di Ricovero e Cura a Carattere Scientifico) Istituto Nazionale Tumori (INT), Milan, 20133 Italy; 147 University of Hawaii Cancer Center, Honolulu, 96813 Hawaii USA; 148 Department of Oncology - Pathology, Karolinska Institutet, Stockholm, SE-171 77 Sweden; 149 National Center for Tumour Diseases, University of Heidelberg, Heidelberg, 69117 Germany; 150 Department of Gynaecology, Radboud University Medical Centre, Nijmegen, 6500 The Netherlands; 151 Department of Preventive Medicine, Kyushu University Faculty of Medical Sciences, Fukuoka, 812-8582 Japan; 152 Anatomical Pathology, The Alfred Hospital, Melbourne, 3004 Victoria Australia; 153 Institute of Cancer Sciences, University of Glasgow, Wolfson Wohl Cancer Research Centre, Beatson Institute for Cancer Research, Glasgow, G61 1BD UK; 154 Division of Gynaecology and Obstetrics, Technische Universität München, Munich, 81675 Germany; 155 Department of Human Genetics, Radboud University Medical Centre, Nijmegen, 6500 The Netherlands; 156 Immunology and Molecular Oncology Unit, Instituto Oncologico Veneto IOV, IRCCS, Padua, 35128 Italy; 157 Department of Cancer Prevention and Control, Roswell Park Cancer Institute, Buffalo, 14263 New York USA; 158 Institute of Population Health, University of Manchester, Manchester, M13 9PL UK; 159 Laboratory Medicine Program, University Health Network, Toronto, M5G 1L7 Ontario Canada; 160 Department of Laboratory Medicine and Pathobiology, University of Toronto, Toronto, M5G 1L7 Ontario Canada; 161 The University of Texas School of Public Health, Houston, 77030 Texas USA; 162 Department of Population Sciences, Beckman Research Institute of City of Hope, Duarte, 91010 California USA; 163 Department of Medicine and Genetics, University of California, San Francisco, 94143 California USA; 164 Department of Gynecological Oncology, Roswell Park Cancer Institute, Buffalo, 14263 New York USA; 165 Department of Medicine, Memorial Sloan-Kettering Cancer Center, New York, 10065 USA; 166 Department of Molecular Genetics, National Institute of Oncology, Budapest, 1122 Hungary; 167 Center for Clinical Cancer Genetics and Global Health, University of Chicago Medical Center, Chicago, 60637 Illinois USA; 168 The Ohio State University and the James Cancer Center, Columbus, 43210 Ohio USA; 169 Department of Epidemiology and Biostatistics, Memorial Sloan Kettering Cancer Center, New York, 10017 USA; 170 Human Genetics Group, Human Cancer Genetics Program, Spanish National Cancer Centre (CNIO), Madrid, 28019 Spain; 171 Biomedical Network on Rare Diseases (CIBERER), Madrid, 28029 Spain; 172 Department of Surgery, Seoul National University College of Medicine, Seoul, 03080 Korea; 173 Department of Obstetrics and Gynecology, Oregon Health and Science University, Portland, 97239 Oregon USA; 174 Knight Cancer Institute, Oregon Health and Science University, Portland, 97239 Oregon USA; 175 IFOM, The FIRC (Italian Foundation for Cancer Research) Institute of Molecular Oncology, Milan, 16 20139 Italy; 176 Department of Obstetrics and Gynecology, Comprehensive Cancer Center, Medical University of Vienna, Vienna, 1090 Austria; 177 Department of Cancer Epidemiology, Moffitt Cancer Center, Tampa, 33606 Florida USA; 178 Channing Division of Network Medicine, Brigham and Women's Hospital and Harvard Medical School, Boston, 02115 Massachusetts USA; 179 Department of Epidemiology, Harvard TH Chan School of Public Health, Boston, 02115 Massachusetts USA; 180 Laboratory of Cancer Genetics and Tumour Biology, Northern Finland Laboratory Centre NordLab, Oulu, FI-90014 Finland; 181 Department of Clinical Chemistry and Biocenter Oulu, Laboratory of Cancer Genetics and Tumour Biology, University of Oulu, Oulu, FI-90014 Finland; 182 Department of Preventive and Predictive Medicine, Unit of Molecular Bases of Genetic Risk and Genetic Testing, Fondazione IRCCS (Istituto Di Ricovero e Cura a Carattere Scientifico) Istituto Nazionale dei Tumori (INT), Milan, 20133 Italy; 183 Department of Clinical Genetics, Karolinska University Hospital, Stockholm, 171 76 Sweden; 184 Department of Basic Sciences, Shaukat Khanum Memorial Cancer Hospital and Research Centre (SKMCH & RC), Lahore, 54000 Pakistan; 185 Clalit National Israeli Cancer Control Center and Department of Community Medicine and Epidemiology, Carmel Medical Center and B. Rappaport Faculty of Medicine, Haifa, 34362 Israel; 186 Department of Gynaecology and Obstetrics and Centre for Integrated Oncology (CIO), Centre of Familial Breast and Ovarian Cancer, Center for Molecular Medicine Cologne (CMMC), University Hospital of Cologne, Cologne, 50931 Germany; 187 Department of Chronic Disease Epidemiology, Yale School of Public Health, New Haven, 06510 Connecticut USA; 188 Division of Gynecologic Oncology, NorthShore University HealthSystem, Evanston, 60201 Illinois USA; 189 Division of Public Health Sciences, Program in Epidemiology, Fred Hutchinson Cancer Research Center, Seattle, 98109 Washington USA; 190 Department of Epidemiology, University of Washington, Seattle, 98109 Washington USA; 191 National Cancer Institute, Bangkok, 10400 Thailand; 192 Research Oncology, Guy’s Hospital, King's College London, London, SE1 9RT UK; 193 Department of Community and Family Medicine, Duke University Medical Center, Durham, 27710 North Carolina USA; 194 Cancer Control and Population Sciences, Duke Cancer Institute, Durham, 27710 North Carolina USA; 195 Netherlands Cancer Institute, Antoni van Leeuwenhoek Hospital, Amsterdam, 1066 CX The Netherlands; 196 Division of Molecular Gyneco-Oncology, Department of Gynaecology and Obstetrics, University Hospital of Cologne, Cologne, 50676 Germany; 197 Center for Integrated Oncology, University Hospital of Cologne, Cologne, 50676 Germany; 198 Center for Molecular Medicine, University Hospital of Cologne, Cologne, 50676 Germany; 199 Center of Familial Breast and Ovarian Cancer, University Hospital of Cologne, Cologne, 50676 Germany; 200 Taiwan Biobank, Institute of Biomedical Sciences, Academia Sinica, Taipei, 115 Taiwan; 201 School of Public Health, China Medical University, Taichung, 404 Taiwan; 202 Department of Health Research and Policy - Epidemiology, Stanford University School of Medicine, Stanford, 94305 California USA; 203 Unité Mixte de Génétique Constitutionnelle des Cancers Fréquents, Hospices Civils de Lyon – Centre Léon Bérard, Lyon, 69008 France; 204 INSERM U1052, CNRS UMR5286, Université Lyon 1, Centre de Recherche en Cancérologie de Lyon, Lyon, 69003 France; 205 Department of Pathology, University of Melbourne, Parkville, 3010 Victoria Australia; 206 Division of Clinical Genetics, Department of Clinical and Experimental Medicine, Linköping University, Linköping, 581 83 Sweden; 207 Institut Curie, Department of Tumour Biology, Paris, France; Department of Tumour Biology, Institut Curie, Paris, France, ,; 311 Institut Curie, INSERM U830, Paris, 75248 France; 208 Université Paris Descartes, Sorbonne Paris Cité, Paris, 75270 France; 209 Department of Human Genetics, Institute of Human Genetics, University Hospital Heidelberg, Heidelberg, 69120 Germany; 210 Department of Genetics, Portuguese Oncology Institute, Porto, 4200-072 Portugal; 211 Biomedical Sciences Institute (ICBAS), Porto University, Porto, 4099-002 Portugal; 212 Department of Epidemiology, Mailman School of Public Health, Columbia University, New York, 10027 USA; 213 Department of Clinical Genetics, Odense University Hospital, Odense C, 5000 Denmark; 214 UO Anatomia Patologica, Ospedale di Circolo-Università dell'Insubria, Varese, 21100 Italy; 215 Latvian Biomedical Research and Study Centre, Riga, LV-1067 Latvia; 216 Immunology and Molecular Oncology Unit, Istituto Oncologico Veneto IOV - IRCCS (Istituto Di Ricovero e Cura a Carattere Scientifico), Padua, 64 - 35128 Italy; 217 Department of Molecular Virology, Immunology and Medical Genetics, The Ohio State University, Columbus, 43210 Ohio USA; 218 Wellcome Trust Centre for Human Genetics and Oxford Biomedical Research Centre, University of Oxford, Oxford, OX3 7BN UK; 219 Institute of Human Genetics, Pontificia Universidad Javeriana, Cra. 7 #40-62, Bogota, Colombia; 220 Department of Medical Oncology, Beth Israel Deaconess Medical Center, Boston, 02215 Massachusetts USA; 221 Department of Clinical Genetics, Erasmus University Medical Center, Rotterdam, 3015 CE The Netherlands; 222 Department of Gynecology, Family Cancer Clinic, Erasmus MC Cancer Institute, Rotterdam, 3015 CE The Netherlands; 223 Division of Gynecological Oncology, Department of Oncology, University Hospitals Leuven, Leuven, B-3000 Belgium; 224 university hospital ulm, Ulm, 89069 Germany; 225 Division of Cancer Epidemiology and Genetics, National Cancer Institute, Bethesda, 20892 Maryland USA; 226 Department of General Medical Oncology, Multidisciplinary Breast Center, University Hospitals Leuven, Leuven, B-3000 Belgium; 227 Molecular Diagnostics Laboratory, IRRP, National Centre for Scientific Research ‘Demokritos’, Athens, 153 10 Greece; 228 Cancer Research Initiatives Foundation, Sime Darby Medical Centre, Subang Jaya, 47500 Malaysia; 229 University Malaya Cancer Research Institute, Faculty of Medicine, University Malaya Medical Centre, University Malaya, Kuala Lumpur, 59100 Malaysia; 230 Department of Surgery, Tri-Service General Hospital, National Defense Medical Center, Taipei, 114 Taiwan; 231 Shanghai Center for Disease Control and Prevention, Shanghai, China; 232 Division of Population Sciences, Cancer Epidemiology Program, H. Lee Moffitt Cancer Center & Research Institute, Tampa, 33612 Florida USA; 233 Department of Laboratory Medicine and Pathology, Mayo Clinic, Rochester, 55905 Minnesota USA; 234 Peter MacCallum Cancer Centre, East Melbourne, 3002 Victoria Australia; 235 Sir Peter MacCallum Cancer Centre Department of Oncology, University of Melbourne, Parkville, 3052 Victoria Australia; 236 Department of Surgery and Cancer, Ovarian Cancer Action Research Centre, Imperial College London, London, W12 0HS UK; 237 Department of Biochemistry and Molecular Biology, University of Melbourne, Parkville, 3052 Victoria Australia; 238 Department of Gynaecological Oncology, Westmead Institute for Cancer Research, Westmead Hospital, Westmead, 2145 New South Wales Australia; 239 Service de Génétique, CHU de Besançon, Besançon, 25030 France; 240 Oncogénétique, Institut Bergonié, 229 cours de l'Argonne, Bordeaux, 33076 France; 241 Centre François Baclesse, 3 avenue Général Harris, Caen, 14000 France; 242 Laboratoire de Génétique Chromosomique, Hôtel Dieu Centre Hospitalier, Chambéry, BP 1125 France; 243 Centre Jean Perrin, Clermont-Ferrand cedex, BP 392 France; 244 Centre de Lutte Contre le Cancer Georges François Leclerc, 1 rue Professeur Marion, Dijon Cedex, BP 77 980 France; 245 Département de Génétique, CHU de Grenoble, Grenoble Cedex 9, BP 217 France; 246 Centre Oscar Lambret, 3 rue Frédéric Combemale, 59020, Lille cedex, BP307 France; 247 Department of Medical Oncology, CHU Dupuytren, Limoges, 87042 France; 248 Service de Génétique Moléculaire et Clinique, Hôpital Edouard Herriot, 5 place d'Arsonval, Lyon cedex 03, 69437 France; 249 Centre Léon Bérard, 28 rue Laënnec, Lyon, 69437 France; 250 Unité de Prévention et d’Epidémiologie Génétique, Centre Léon Bérard, 28 rue Laënnec, Lyon, 69437 France; 251 Biopathologie, Centre Léon Bérard, 28 rue Laënnec, Lyon, 69437 France; 252 Département Oncologie Génétique, Prévention et Dépistage, Institut Paoli-Calmettes, 232 boulevard Sainte-Marguerite, Marseille, 13009 France; 253 Unité d'Oncogénétique, CHU Arnaud de Villeneuve, Montpellier Cedex 5, 34295 France; 254 Laboratoire de génétique médicale, Nancy Université, Centre Hospitalier Régional et Universitaire, Rue du Morvan, cedex 1, 54511 Vandoeuvre-les-Nancy France; 255 Service d'Oncogénétique, Centre René Gauducheau, Boulevard Jacques Monod, Nantes Saint Herblain Cedex, 44805 France; 256 Centre Antoine Lacassagne, 33 Avenue de Valombrose, Nice, 06100 France; 257 Service de Génétique, Institut Curie, 26, rue d’Ulm, Paris Cedex 05, 75248 France; 258 Inserm U830, Université INSERM U830, centre de recherche de l'Institut Curie, Paris, 75013 France; 259 Inserm U900, Institut Curie, Mines ParisTech, PSL University, 26 rue d'Ulm, Paris Cedex 05, 75248 France; 260 Département de Génétique, Groupe Hospitalier Pitié-Salpétrière, 47-83 boulevard de l'Hôpital, Paris, 75013 France; 261 Service de Génétique Clinique Chromosomique et Moléculaire, Hôpital Nord, CHU Saint Etienne, St Etienne, 42055 Cedex 2 France; 262 Unité d’Oncogénétique, Centre Paul Strauss, 3 rue de la Porte de l'Hôpital, Strasbourg, BP30042 France; 263 Oncogénétique, Institut Claudius Regaud, 1 avenue Irène Joliot-Curie, Toulouse cedex 9, 31059 France; 264 Hôpital Bretonneau - CHU de Tours, 2 boulevard Tonnelé, Tours cedex, 37004 France; 265 Service de Génétique, Institut Gustave Roussy, 39, rue Camille Desmoulins, Villejuif Cedex, 94805 France; 266 North of Scotland Regional Genetics Service, NHS Grampian & University of Aberdeen, Foresterhill, AB24 3AA Aberdeen UK; 267 and Department of Medical Genetics, Northern Ireland Regional Genetics Centre, Belfast Health and Social Care Trust, Queens University Belfast, Belfast, BT9 7BL UK; 268 Clinical Genetics Department, St Michael’s Hospital, Bristol, BS2 8EG UK; 269 All Wales Medical Genetics Services, University Hospital of Wales, Cardiff, CF14 4XW UK; 270 Academic Unit of Clinical and Molecular Oncology, Trinity College Dublin, Dublin, 2 Ireland; 271 St James's Hospital, Dublin, 8 Ireland; 272 South East of Scotland Regional Genetics Service, Western General Hospital, Edinburgh, EH4 2XU UK; 273 North West Thames Regional Genetics Service, Kennedy-Galton Centre, Harrow, HA1 3UJ UK; 274 Leicestershire Clinical Genetics Service, University Hospitals of Leicester NHS Trust, Leicester, LE1 5WW UK; 275 Department of Clinical Genetics, Alder Hey Hospital, Eaton Road, Liverpool, L12 2AP UK; 276 Genetic Medicine, Manchester Academic Health Sciences Centre, Central Manchester University Hospitals NHS Foundation Trust, Manchester, M13 9WL UK; 277 North East Thames Regional Genetics Service, Great Ormond Street Hospital for Children NHS Trust, London, WC1N 3JH UK; 278 Nottingham Clinical Genetics Service, Nottingham University Hospitals NHS Trust, Nottingham, NG5 1PB UK; 279 Institute of Genetic Medicine, Centre for Life, Newcastle Upon Tyne Hospitals NHS Trust, Newcastle upon Tyne, NE7 7DN UK; 280 Oxford Regional Genetics Service, Churchill Hospital, Oxford, OX3 7LE UK; 281 Sheffield Clinical Genetics Service, Sheffield Children’s Hospital, Sheffield, S10 2TH UK; 282 South West Thames Regional Genetics Service, St.Georges Hospital, Cranmer Terrace, Tooting, London, SW17 0RE UK; 283 All Wales Medical Genetics Services, Singleton Hospital, Swansea, SA2 8QA UK; 284 All Wales Medical Genetics Service, Glan Clwyd Hospital, Rhyl, LL18 5UJ UK; 285 Department of Epidemiology, Netherlands Cancer Institute, PO Box 90203, Amsterdam, 1006 BE The Netherlands; 286 Family Cancer Clinic, Netherlands Cancer Institute, PO Box 90203, Amsterdam, 1006 BE The Netherlands; 287 Division of Psychosocial Research and Epidemiology, Netherlands Cancer Institute, PO Box 90203, Amsterdam, 1006 BE The Netherlands; 288 Department of Radiotherapy, Netherlands Cancer Institute, PO Box 90203, Amsterdam, 1006 BE The Netherlands; 289 Department of Clinical Genetics, Family Cancer Clinic, Erasmus University Medical Center, PO Box 2040, Amsterdam, 3000 CA The Netherlands; 290 Department of Medical Oncology, Family Cancer Clinic, Erasmus MC Cancer Institute, PO Box 5201, Rotterdam, 3008 AE The Netherlands; 291 Department of Pathology, Family Cancer Clinic, Erasmus University Medical Center, PO Box 2040, Amsterdam, 3000 CA The Netherlands; 292 Department of Radiology, Family Cancer Clinic, Erasmus University Medical Center, PO Box 2040, Amsterdam, 3000 CA The Netherlands; 293 Department of Clinical Genetics, Leiden University Medical Center, PO Box 9600, Leiden, 2300 RC The Netherlands; 294 Department of Surgery, Leiden University Medical Center, PO Box 9600, Leiden, 2300 RC The Netherlands; 295 Department of Human Genetics, Radboud University Medical Center, PO Box 9101, Nijmegen, 6500 HB The Netherlands; 296 Department of Medical Genetics, University Medical Center Utrecht, Utrecht, PO Box 85090, 3508 AB The Netherlands; 297 Department of Oncological and Endocrine Surgery, University Medical Center Utrecht, PO Box 85090, Utrecht, 3508 AB The Netherlands; 298 Department of Clinical Genetics, Academic Medical Center, PO Box 22700, Amsterdam, 1100 DE The Netherlands; 299 Department of Clinical Genetics, VU University Medical Center, PO Box 7057, Amsterdam, 1007 MB The Netherlands; 300 Department of Clinical Genetics and GROW, School for Oncology and Developmental Biology, Maastricht University Medical Center, PO Box 5800, Maastricht, 6202 AZ The Netherlands; 301 Department of Genetics, University Medical Center Groningen, PO Box 30.001, Groningen, 9700 RB The Netherlands; 302 Department of Gynaecology, University Medical Center Groningen, PO Box 30.001, Groningen, 9700 RB The Netherlands; 303 Department of Epidemiology, University Medical Center Groningen, PO Box 30.001, Groningen, 9700 RB The Netherlands; 304 The Netherlands Foundation for Detection of Hereditary Tumours, University Medical Center, Poortgebouw Zuid, Leiden, 2333 AA The Netherlands; 305 The Netherlands Comprehensive Cancer Organization (IKNL), Location Amsterdam, IJsbaanpad 9-11, Amsterdam, 1076 CV The Netherlands; 306 The nationwide network and registry of histo- and cytopathology in the Netherlands (PALGA), Randhoeve 225A, Houten, 3995 GA The Netherlands; 307 Pathology Department, Peter MacCallum Cancer Centre, Melbourne, 3002 Victoria Australia; 308 Department of Medicine, Familial Cancer Service, Westmead Hospital, Westmead, 2145 New South Wales Australia; 309 Breast Cancer Laboratory, Walter and Eliza Hall Institute, PO Royal Melbourne Hospital, Parkville, 3050 Victoria Australia; 310 Medical Psychology, University of Sydney, Sydney, 2006 New South Wales Australia; 314 Present Address: Present address: Women's Cancer Program at the Samuel Oschin Comprehensive Cancer Institute, Cedars-Sinai Medical Center, Los Angeles, CA, USA, ,; 315 Present Address: Present address: The Center for Bioinformatics and Functional Genomics, Cedars-Sinai Medical Center, Los Angeles, CA, USA, ,

**Keywords:** Cancer genetics, Genetic association study, Epigenetics, Breast cancer

## Abstract

A locus at 19p13 is associated with breast cancer (BC) and ovarian cancer (OC) risk. Here we analyse 438 SNPs in this region in 46,451 BC and 15,438 OC cases, 15,252 *BRCA1* mutation carriers and 73,444 controls and identify 13 candidate causal SNPs associated with serous OC (*P*=9.2 × 10^−20^), ER-negative BC (*P*=1.1 × 10^−13^), *BRCA1*-associated BC (*P*=7.7 × 10^−16^) and triple negative BC (*P*-diff=2 × 10^−5^). Genotype-gene expression associations are identified for candidate target genes *ANKLE1* (*P*=2 × 10^−3^) and *ABHD8* (*P*<2 × 10^−3^). Chromosome conformation capture identifies interactions between four candidate SNPs and *ABHD8*, and luciferase assays indicate six risk alleles increased transactivation of the *ADHD8* promoter. Targeted deletion of a region containing risk SNP rs56069439 in a putative enhancer induces *ANKLE1* downregulation; and mRNA stability assays indicate functional effects for an *ANKLE1* 3′-UTR SNP. Altogether, these data suggest that multiple SNPs at 19p13 regulate *ABHD8* and perhaps *ANKLE1* expression, and indicate common mechanisms underlying breast and ovarian cancer risk.

**Supplementary information:**

The online version of this article (doi:10.1038/ncomms12675) contains supplementary material, which is available to authorized users.

## Abstract (web summary)

A region on chromosome 19p13 is associated with the risk of developing ovarian and breast cancer. Here, the authors genotyped SNPs in this region in thousands of breast and ovarian cancer patients and identified SNPs associated with three genes, which were analysed with functional studies.

**Supplementary information:**

The online version of this article (doi:10.1038/ncomms12675) contains supplementary material, which is available to authorized users.

## Graphical abstract

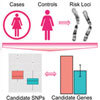

## Introduction

Genome-wide association studies (GWAS) have identified more than 100 different genetic susceptibility regions for breast cancer (BC)^1,2,3,4,5,6^ and 20 regions for epithelial ovarian cancer (EOC)^7,8,9,10,11,12,13^. A few of these regions, and in some cases the same genetic variants, are associated with risks of both cancers (pleiotropy), suggesting there may be underlying functional mechanisms and biological pathways common to different cancers. The *TERT-CLPTM1L* locus (5p15) is one such example in which the same variants are associated with risks of oestrogen receptor (ER)-negative BC, BC in BRCA1 mutation carriers and serous invasive OC^10^.

Few studies have comprehensively described the functional mechanisms underlying common variant susceptibility loci^10,14,15,16,17,18^. More than 90% of risk alleles lie in non-protein-coding DNA and there is now unequivocal evidence that susceptibility regions are enriched for risk-associated single-nucleotide polymorphisms (SNPs) intersecting regulatory elements, such as transcriptional enhancers, predicted to control the expression of target genes *in cis*^19,20,21^. Establishing causality for risk SNPs is very challenging; of the thousands of risk associations identified by GWAS, functional validation of causal variants using genome editing has only been experimentally performed for two SNPs, one for prostate cancer^22^ using the CAUSEL pipeline and the other for obesity^23^. Thus, there is a critical need to identify the causal risk SNP(s) and the overlapping regulatory element(s) and the target gene(s) regulated in an allele-specific manner.

Breast and high-grade serous OC share common genetic and non-genetic risk factors, with mutations in *BRCA1* and *BRCA2* the most significant risk factors for both cancers, suggesting similar biological mechanisms drive breast and OC development. A region on chromosome 19p13.1 has previously been associated with susceptibility to BC and OC in the general population, and to modify the risks of *BRCA1*-related BC and *BRCA2*-related OC^9,24,25,26,27^. Initial studies indicated that the association signal was centred around the SNP rs8170 located in the *BRCA1*-interacting gene *BABAM1* (ref. 9), and subsequent studies have refined the subtype specific BC risks associated with these SNPs^24,25,26,28^.

In the current study, we hypothesized that the same functional mechanism underlies the 19p13.1 risk association in both BC and OC. To evaluate this hypothesis we performed genetic fine mapping in BC and OC patients and in *BRCA1* mutation carriers, and performed a wide range of functional assays in breast and ovarian tissues and *in vitro* models to identify the likely causal alleles, and target regulatory elements and susceptibility gene(s). Our data indicate that multiple SNPs are involved in the regulation of *ABHD8* and perhaps *ANKLE1* at this locus.

## Results

### Genetic association analyses with breast and OC risks

**Figure 1:**
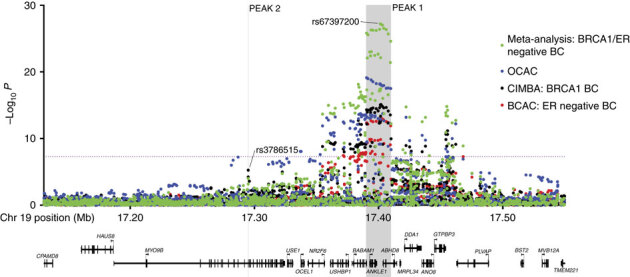
Regional association plot disease-specific risk associations. Results for ER negative breast cancer from BCAC, for ovarian cancer from OCAC and for *BRCA1* mutation carriers with breast cancer from CIMBA are shown. Also shown are the results of a meta-analysis for BRCA1 and general population ER negative breast cancer cases. The grey bars indicate the boundaries of the two association peaks, and the dotted horizontal line indicates the cutoff for genome-wide significance (*χ*^2^-test *P*=5 × 10^−8^). Previously identified GWAS SNPs are indicated with italic font. Genes in the region are displayed beneath the association results.

A total of 438 SNPs spanning 420 kb at the chromosome 19p13 locus (nucleotides 17,130,000–17,550,000 (NCBI build 37)) were genotyped successfully in the following populations: 46,451 BC cases (of which 7,435 cases had ER-negative tumours) and 42,599 controls from the Breast Cancer Association Consortium (BCAC); 15,438 cases of EOC (of which 9,630 were of serous histology) and 30,845 controls from the Ovarian Cancer Association Consortium (OCAC); and 15,252 *BRCA1* mutation carriers from the Consortium of Investigators of Modifiers of *BRCA1/2* (CIMBA; 7,797 with BC and 7,455 unaffected; Supplementary Table 1). Genotypes for variants identified through the 1,000 genomes project (minor allele frequency (MAF)>0.1%) were imputed for all participants of European ancestry. A total of 2,269 genotyped and imputed SNPs were analysed for their associations with ER-negative BC risk in the general population, 2,311 SNPs with BC/OC risk for *BRCA1* mutation carriers, and 2,565 SNPs with risk of serous OC. Results for all SNPs associated with these phenotypes at *P*<10^−4^ are illustrated in Fig. 1 and Supplementary Fig. 1. Two perfectly correlated SNPs rs61494113 and rs67397200 located between the *ANKLE1* and *ABHD8* genes demonstrated the strongest association with BC risk among *BRCA1* mutation carriers (*χ*^2^-test *P*=7.8 × 10^−16^) and ER-negative BC in BCAC (*χ*^2^-test *P*=1.3 × 10^−13^, *P*-meta-analysis=7.3 × 10^−28^). There was no association for ER-positive BC (*χ*^2^-test *P*=0.21 for rs61494113). The strongest association with invasive and serous OC was for rs4808075 (correlated with rs61494113 with *r*^2^=0.99) located in the *BABAM1* gene (*χ*^2^-test *P*=9.2 × 10^−20^). We observed no associations with risk of other histological subtypes of invasive OC (Supplementary Table 2). The correlations between the SNP exhibiting the strongest risk association (rs67397200) in the meta-analysis of BC risk for *BRCA1* mutation carriers and ER-negative BC, with the previously reported risk-associated SNPs for breast, OC and *BRCA1*-associated BCs can be found in Supplementary Table 3.

All SNPs with an association *P* value<0.001 with each phenotype were included in forward stepwise Cox regression models for risks of *BRCA1* BC, and logistic regression models for ER-negative BC and serous OC. The most parsimonious models for ER-negative BC and serous OC each included one SNP, rs67397200 for ER-negative BC and rs4808075 for serous OC (referred to as Peak 1). The most parsimonious model in the analysis of BC risk for *BRCA1* mutation carriers included two virtually uncorrelated SNPs (pairwise correlation *r*^2^=0.018) rs61494113 (*P* value=4.4 × 10^−16^ in conditional regression analysis), and rs3786515 (Peak 2, conditional regression *P* value=9.6 × 10^−5^, pairwise correlation *r*^2^=0.018; Fig. 1). No other SNP was retained in the model at the *P* value threshold of 0.0001.

### Candidate causal variants

Peak 1 includes SNPs that encompass the *BABAM1, ABHD8* and *ANKLE1* gene and are associated with serous OC, ER-negative BC and BC risk for *BRCA1* mutation carriers (Fig. 1 and Supplementary Fig. 1); Peak 2 includes SNPs located in the *MYO9B* gene associated only with BC risk in *BRCA1* mutation carriers. SNPs in Peaks 1 and 2 are virtually uncorrelated.

To identify the strongest candidate causal SNPs, we computed likelihood ratios of each SNP relative to the SNP with the strongest association in each peak for risks of each phenotype. Due to the similarities in associations between ER-negative BC and *BRCA1-*associated BC in Peak 1, we computed the likelihood ratios on the basis of the meta-analysis results. Table 1 includes the SNPs that cannot be excluded at a likelihood ratio of >1:100 fold. In Peak 1, all but 12 SNPs can be excluded from being causal for ER-negative BC and *BRCA1-*associated BC. An additional SNP (rs10424198) cannot be excluded from being causal for serous OC. All 13 SNPs were highly correlated (*r*^2^>0.95) and spanned a region of 19.4 kb. In Peak 2, the likelihood ratios of each SNP were calculated on the basis of the *BRCA1* association analysis conditional on the top SNP rs61494113. All but seven SNPs correlated with rs3786515 (*r*^2^>0.10) cannot be excluded from being the causal SNP for *BRCA1-*associated BC risk. With the exception of rs3786514 (pairwise *r*^2^ with rs3786515=0.87) all other SNPs had *r*^2^ with rs3786515 between 0.13 and 0.20.

**Table 1:** SNPs associated with risk ovarian cancer, ER-negative breast cancer or breast cancer in *BRCA1* carriers at the 19p13 locus.

**SNP** ^*^	**Nucleotide position (build 37)**	**Allele freq.**	**BRCA1 breast cancer**		**ER-negative breast cancer**		***BRCA1*** **/ER-negative breast cancer meta-analysis (** ***P*** **value)**	**Serous ovarian cancer**	
			**HR (95% confidence intervals)**	***P*** **value**	**OR (95% confidence intervals)**	***P*** **value**		**OR (95% confidence intervals)**	***P*** **value**
*Peak 1*									
rs4808075 *(I)*	17390291	0.30	1.19 (1.14–1.24)	4.77 × 10^−15^	1.16 (1.11–1.21)	4.42 × 10^−13^	1.55 × 10^−26^	1.19 (1.14–1.23)	9.17 × 10^−20^
rs10419397 *(I)*	17391328	0.30	1.19 (1.14–1.24)	5.55 × 10^−15^	1.16 (1.11–1.21)	6.57 × 10^−13^	2.7 × 10^−26^	1.19 (1.14–1.23)	1.29 × 10^−19^
rs56069439 *(I)*	17393925	0.30	1.19 (1.14–1.24)	3.33 × 10^−15^	1.16 (1.12–1.21)	2.22 × 10^−13^	5.26 × 10^−27^	1.19 (1.14–1.23)	1.94 × 10^−19^
rs4808076 *(I)*	17395401	0.30	1.19 (1.14–1.24)	2.55 × 10^−15^	1.16 (1.12–1.21)	2.9 × 10^−13^	5.59 × 10^−27^	1.18 (1.14–1.23)	3.72 × 10^−19^
rs111961716 *(I)*	17398085	0.30	1.19 (1.14–1.24)	3.22 × 10^−15^	1.16 (1.12–1.21)	2.63 × 10^−13^	6.07 × 10^−27^	1.18 (1.14–1.23)	6.97 × 10^−19^
rs113299211 *(I)*	17400765	0.30	1.19 (1.14–1.24)	2.33 × 10^−15^	1.16 (1.12–1.21)	2.4 × 10^−13^	4.22 × 10^−27^	1.18 (1.14–1.23)	8.13 × 10^−19^
rs67397200 *(G)*	17401404	0.30	1.19 (1.14–1.24)	8.88 × 10^−16^	1.16 (1.12–1.21)	1.10 × 10^−13^	6.18 × 10^−28^	1.18 (1.14–1.23)	7.75 × 10^−19^
rs61494113 *(G)*	17401859	0.30	1.19 (1.14–1.25)	7.77 × 10^−16^	1.16 (1.12–1.21)	1.27 × 10^−13^	7.31 × 10^−28^	1.18 (1.14–1.23)	1.14 × 10^−18^
rs4808616 *(G)*	17403033	0.31	1.19 (1.14–1.24)	1.44 × 10^−15^	1.16 (1.12–1.21)	1.10 × 10^−13^	9.37 × 10^−28^	1.18 (1.14–1.23)	1.51 × 10^−18^
rs55924783 *(I)*	17404072	0.30	1.19 (1.14–1.24)	2.44 × 10^−15^	1.16 (1.12–1.21)	1.61 × 10^−13^	2.81 × 10^−27^	1.18 (1.14–1.23)	1.35 × 10^−18^
rs28473003 *(I)*	17406167	0.30	1.19 (1.14–1.24)	2.11 × 10^−15^	1.16 (1.12–1.21)	2.8 × 10^−13^	4.55 × 10^−27^	1.18 (1.14–1.22)	3.43 × 10^−18^
rs13343778 *(I)*	17407695	0.30	1.19 (1.14–1.24)	7.44 × 10^−15^	1.16 (1.12–1.21)	3.92 × 10^−13^	2.06 × 10^−26^	1.18 (1.14–1.22)	3.18 × 10^−18^
rs10424198 *(I)*	17409671	0.30	1.18 (1.13–1.24)	3.13 × 10^−14^	1.16 (1.12–1.20)	1.18 × 10^−12^	2.56 × 10^−25^	1.18 (1.14–1.22)	3.85 × 10^−18^
*Peak 2*									
rs3786514 *(G)*	17294954	0.48	1.08 (1.04–1.13)	5.85 × 10^−05^	1.02 (0.98–1.06)	0.364	6.52 × 10^−04^	1.05 (1.01–1.08)	8.01 × 10^−03^
rs3786515 *(G)*	17295023	0.45	1.10 (1.05–1.14)	5.42 × 10^−06^	1.02 (0.98–1.06)	0.281	9.94 × 10^−05^	1.05 (1.01–1.09)	5.62 × 10^−03^
rs891205 *(G)*	17354586	0.61	1.09 (1.05–1.13)	4.16 × 10^−05^	1.05 (1.01–1.09)	0.0164	5.39 × 10^−06^	1.07 (1.04–1.11)	1.26 × 10^−04^
rs7247493 *(G)*	17362941	0.60	1.09 (1.04–1.13)	5.85 × 10^−05^	1.05 (1.01–1.09)	0.014	5.73 × 10^−06^	1.07 (1.04–1.11)	9.68 × 10^−05^
rs7246243 *(I)*	17363068	0.60	1.09 (1.04–1.13)	5.28 × 10^−05^	1.05 (1,01–1.09)	0.0149	5.74 × 10^−06^	1.08 (1.04–1.11)	3.73 × 10^−05^
rs4464206 *(G)*	17367585	0.62	1.10 (1.05–1.14)	7.28 × 10^−05^	1.06 (1.02–1.10)	0.0172	8.87 × 10^−06^	1.08 (1.04–1.12)	2.54 × 10^−05^
C19pos17261271 *(G)*	17400271	0.50	0.92 (0.88–0.96)	2.41 × 10^−05^	0.96 (0.92–0.99)	0.020	4.76 × 10^−06^	0.92 (0.89–0.96)	9.26 × 10^−06^
*Peak 2 (conditional* *P* *values on top SNP from Peak 1*)									
rs3786514	17294954			1.40 × 10^−03^					
rs3786515	17295023			9.13 × 10^−05^					
rs891205	17354586			0.0107					
rs7247493	17362941			0.0131					
rs7246243	17363068			0.0122					
rs4464206	17367585			115					
c19_pos17261271	17400271			6.31 × 10^−03^					

EOC, epithelial ovarian cancer; ER, oestrogen receptor; freq., frequency; HR, hazards ratio; OR, odds ratio; SNP, single-nucleotide polymorphism.

SNPs in Peak 1 and Peak 2 that cannot be excluded at a likelihood ratio of >1:100 fold relative to the most significant SNP for the meta-analysis and serous EOC (Peak 1) and *BRCA1* association breast cancer for Peak 2.

^*^Imputed (I) or genotyped (G) SNPs.

### Associations for *BRCA1* and *BRCA2* mutation carriers

SNPs in Peak 1 were only associated with risk of ER-negative BC for *BRCA1* mutation carriers and provided no evidence of association with ER-positive BC for *BRCA1*. SNPs in Peak 1 were also associated with OC risk for *BRCA1* mutation carriers. SNPs in Peak 2 were also primarily associated with *BRCA1*-related ER-negative BC but there was no evidence of association with OC risk (Supplementary Table 4). SNPs in peak 1 were not associated with overall risk of BC in *BRCA2* carriers (for example, rs67397200 HR for BC=1.00 (95% confidence interval (CI): 0.93–0.89)); however, SNP rs67397200 showed evidence of association with OC for *BRCA2* mutation carriers (hazards ratio (HR)=1.18, 95% CI: 1.06–1.36, *χ*^2^-test *P*=0.0056). SNPs in peak 2 did not show any evidence of association with breast or OC risk for *BRCA2* mutation carriers.

### Associations with risk among BC subtypes

None of the Peak 1 SNPs were associated with risk of ER-positive BC. When analyses were restricted to triple negative BC, the odds ratio (OR) estimates for SNPs in Peak 1 were larger than the corresponding OR estimates for ER-negative disease (Supplementary Table 4). There was no evidence of association with ER-negative and HER2-positive BC risk, with the association restricted only to triple-negative BC (test of difference between triple-negative versus ER-negative/HER2+, *P*-diff=2.2 × 10^−5^ for SNP rs61494113).

### Analysis in Asian and African ancestry studies

None of the SNPs in the fine-mapping region were associated with ER-negative BC in samples of Asian ancestry after adjusting for multiple testing (*P* values≥0.0018). However, the risk alleles of the 13 candidate causal SNPs in Peak 1 are uncommon in the Asian population (MAF=0.0079–0.011); hence, the power to detect an association was limited and, due to the wide CIs for the estimated ORs for these SNPs, we cannot rule out that the minor allele of these SNPs in Asian subjects is associated with similar level of risk as in Europeans. In samples of African ancestry only rs4808616 (MAF=0.22) showed evidence of association with risk for overall BC or ER-negative disease (OR for BC=1.19, 95% CI:1.02–1.39, *χ*^2^-test *P*=0.03; OR for ER-negative BC=1.59, 95% CI: 1.02–2.49, *χ*^2^-test *P*=0.04).

### Functional characterization of the 19p13.1 region

Functional characterization focused on the 13 candidate causal SNPs for ER-negative and *BRCA1*-associated BC and serous OC in Peak 1, based on the hypothesis that the functional mechanisms mediated by one or more of these SNPs were the same for these phenotypes.

### Genotype-gene expression associations

**Figure 2:**
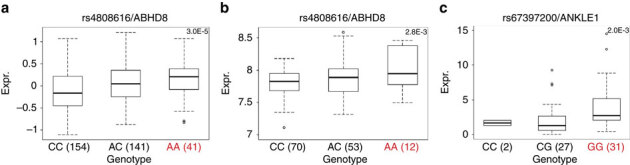
Expression quantitative trait locus analyses. Significant eQTL associations identified between rs4808616 and *ABHD8* expression in (**a**) ovarian cancer tissues and (**b**) in normal breast tissues. (**c**) A significant association was also identified between rs4808616 and *ANKLE1* expression in primary normal ovarian/fallopian tube epithelial cell cultures. The horizontal line indicates the median expression, the limits of the boxes denote the first and third quartiles, and the whiskers represent 1.5 times the interquartile range of the data. Outliers are indicated with circles.

We used expression quantitative trait locus (eQTL) analyses to evaluate associations between risk SNPs and the expression of genes in a 1 Mb region spanning rs4808075 in: 135 normal breast tissues^29^, 60 normal ovarian and fallopian tube epithelial cell cultures, 391 ER positive BCs^30^, 59 ER-negative BCs^29^ and 340 high-grade serous OCs^30^. We identified significant eQTL associations for *ABHD8* expression (linear regression *P* value range 2 × 10^−3^–7 × 10^−3^) in normal breast tissues and between rs480816 and *ABHD8* expression in OCs (linear regression *P*=3 × 10^−5^). In both instances the risk allele was associated with higher *ABHD8* expression (Fig. 2a, Supplementary Data 1 and 2 and Supplementary Table 5). We examined whether risk SNPs were the top eQTL SNPs in this region. rs4808616 was the strongest predictor of *ABHD8* expression in OCs. However, in normal breast tissues the top eQTL SNP for *ABHD8* was rs11666308 (linear regression *P*=3.3 × 10^−4^), a marginally better predictor than rs4808616 (linear regression *P*=2.8 × 10^−3^). The two SNPs were correlated (*r*^2^=0.79) and regressing out effects of either SNP from the expression levels of *ABHD8* and repeating eQTL analysis abolished the eQTL signal for the other SNP, confirming their statistical inseparability. In addition we found significant associations between rs4808616 and *NXNL1* expression in OCs (linear regression *P*=4 × 10^−3^) and with *ANKLE1* expression (*P*=0.002) in normal ovarian surface epithelial cells (OSECs). There were no eQTL associations for any other genes in the region.

We also performed allele-specific expression analysis in BC using RNA sequencing data^31^ for coding SNPs in *ABHD8* (rs56069439) and *BABAM1* (rs10424198). Both SNPs were correlated with rs4808616 (*r*^2^=0.91). There was a significant association between rs56069439 and the allelic ratio of *ABHD8* transcripts (F-test *P*=0.016) with greater expression associated the risk allele (Supplementary Fig. 2; Supplementary Data 3).

### Chromosome conformation capture

**Figure 3:**
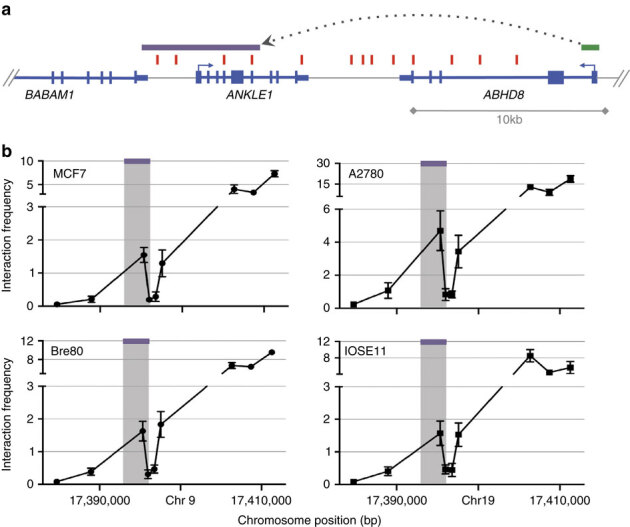
Chromosome conformation capture analysis of long-range interactions at the 19p13 region. 3C interaction profiles in breast and ovarian cell lines. 3C libraries were generated with *Nco*I, with the anchor point set at the *ABHD8* promoter region. (**a**) A physical map of the region interrogated by 3C is shown, with annotated genes shown in blue, the 13 risk-associated SNPs shown in red, the *ABHD8* promoter fragment shown in green and the position of the interacting *Nco*I fragment represented by the purple bar (not to scale). (**b**) Relative interaction frequencies between the *ABHD8* promoter and regions spanning risk associated SNPs in normal breast (Bre80) and ovarian (IOSE11) epithelial cells lines, and in breast (MCF7) and ovarian (A2780) cancer cell lines. A peak of interaction with the *ABHD8* was observed for one region (purple bar) in all four cell lines. There were no interactions detected between the purple region and the *BABAM or USHBP1* promoters. The interacting region contains four candidate causal SNPs (from left to right) rs4808075, rs10419397, rs56069439 and rs4808076. Error bars represent s.d. (*N*=3).

Chromosome conformation capture (3C) analysis was used to investigate DNA–DNA interactions between *ABHD8* and 5 of 13 candidate causal SNPs in Peak 1. Eight SNPs close to the *ABHD8* promoter were too near to be resolved, and the close proximity of candidate causal SNPs to *ANKLE1* precluded 3C analysis for this gene. The *ABHD8* promoter showed an interaction with a 6.3 kb region ∼20 kb telomeric to the gene in both normal breast (Bre80) and ovarian (IOSE11) epithelial cells, and in breast (MCF7) and ovarian (A2780) cancer cell lines (Fig. 3). This region spans the *ANKLE1* promoter and includes four candidate causal SNPs: rs4808075, rs10419397, rs56069439 and rs4808076. There was no evidence of interaction for any candidate causal SNP with *BABAM1* (Supplementary Fig. 3).

### Annotation of candidate causal SNPs

**Figure 4:**
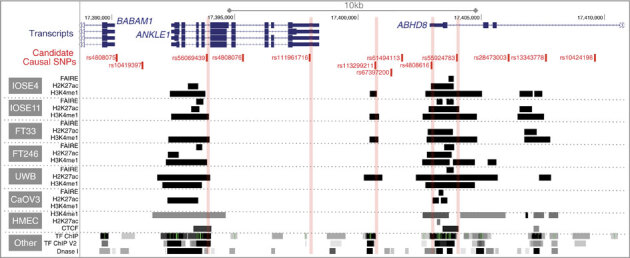
Epigenetic marks intersecting candidate causal SNPs in the 19p13 susceptibility region and analyses of UTR SNPs. The thirteen candidate SNPs were aligned with open chromatin and enhancer marks (H3K27ac and H3K4me1) in high-grade serous ovarian cancer cells (UWB1.289 and CaOV3) and ovarian cancer precursor cells (ovarian epithelial cells, IOSE and fallopian epithelial cells, FT). Enhancer and insulator (CTCF) data for human mammary epithelial cells (HMECs) were obtained from ENCODE. Five SNPs coincide with biofeatures in breast and/or ovarian cells (indicated in red).

All 13 candidate causal SNPs were located in non-protein coding DNA. We annotated putative functional regulatory elements that coincided with the candidate causal SNPs in normal human mammary epithelial cells (HMECs), and normal fallopian tube and ovarian epithelial cells^19^, and in OC cell lines. Five of the 13 SNPs coincide with regulatory elements that were reproducible in two biological replicate samples (Fig. 4). Three SNPs were located in epigenetic marks in breast and/or ovarian cells: rs55924783 coincided with insulator marks in HMECs and enhancer marks in ovarian cells; rs113299211 coincided with enhancer marks in ovarian cells and is predicted to alter transcription factor binding sites for ELF1, ELK4 and GABP; and rs56069439 coincided with experimentally derived ChIP-seq footprints (for CTCF, ATF2 and ZNF263), enhancer marks in ovarian cells and both enhancer (H3K4me1) and insulator (CTCF) marks in breast cells. Two SNPs were located in 3′-untranslated regions (UTRs) of protein coding genes: rs111961716 in *ANKLE1* and rs4808616 in *ABHD8*. rs4808616 also coincided with enhancer marks in ovarian and breast cells. Finally, rs10419397 lay within the putative promoter of *ANKLE1*, ∼1,200 bp from the transcription start site.

### Functional analysis of candidate causal SNPs in UTRs

**Figure 5:**
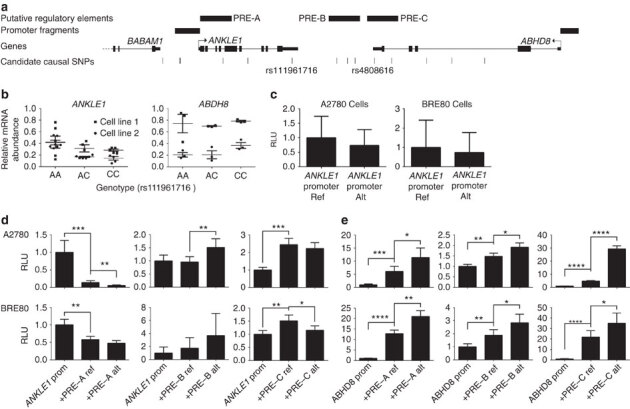
Allele specific analysis of susceptibility SNPs. (**a**) Location of SNPs in putative regulatory elements (PREs) and 5′ untranslated regions. (**b**) RNA stability assays in primary ovarian epithelial cell lines for risk-associated UTR SNPs in *ABHD8* and *ANKLE1*. Normal ovarian epithelial cell lines carrying different genotypes of the risk SNP rs4808616, located in the 3′ UTR of *ABHD8*. Rs4808616 is tightly correlated with rs111961716 (*R*^2^=0.98) located in the 3′ UTR of *ANKLE1*. The risk allele of rs111961716 was associated with decreased mRNA stability of *ANKLE1* compared with the protective allele (*P*=0.006, ANOVA). Different genotypes of rs4808616 are not associated with the stability the *ABHD8* transcript. (**c**–**e**) Luciferase assays to evaluate SNP-dependent promoter and enhancer activity. (**c**) The *ANKLE1* promoter SNP did not affect *ANKLE1* expression in ovarian cancer cells (A2780) and normal breast cells (Bre80). (**d**) Allele-specific activity of PRE-A, PRE-B and PRE-C on the *ANKLE1* promoter. (**e**) Allele-specific activity of PRE-A, PRE-B and PRE-C on *ABHD8* promoter activity. *P>0.05, **P>0.01, ***P>0.001, ****P>0.0001, two-way ANOVA. RLU, relative light units.

We evaluated the effects on mRNA stability of the SNPs located in 3′ UTRs of *ANKLE1* (rs111961716) and *ABHD8* (rs4808616, Figs 4 and 5a) in normal primary ovarian epithelial cell lines carrying different SNP genotypes. RNA transcript abundance was measured after blocking mRNA transcription by treating cells with actinomycin D. For rs111961716, *ANKLE1* transcript expression was significantly more stable in cell lines homozygous for the A (risk) allele of rs111961716 compared with heterozygous cells or cells homozygous for the C allele (*P*=0.006, analysis of variance; Fig. 5b). There was no association between *ABHD8* mRNA stability and genotypes of rs4808616 (Fig. 5b).

### Functional analysis of promoter and enhancer SNPs

Seven of the 13 candidate causal SNPs in Peak 1 resided either in the *ANKLE1* promoter or in putative regulatory elements (PREs-A-C) in breast and ovarian normal and cancer cell lines (Figs 4 and 5a). SNP rs10419397 fell within the *ANKLE* promoter region, but had no effect on promoter activity (Fig. 5c). PRE-A contained SNP rs56069439, PRE-B contained SNPs rs113299211, rs67397200, rs61494113 and PRE-C contained SNPs rs4808616 and rs55924783. We examined the effect of these PREs, and of the risk alleles of each SNP cloned into luciferase constructs containing the *ABHD8* or *ANKLE1* promoters. Inclusion of the reference allele of PREs A, B and C significantly increased *ABHD8* promoter activity in both OC (A2780) and normal breast (Bre80) cell lines (Fig. 5). Constructs containing the risk alleles further enhanced *ABHD8* promoter activity compared with the reference allele for PREs A, B and C in Bre80 cells (*P* values=0.0027, 0.0308 and 0.0342, respectively, two-way analysis of variance (ANOVA)) and for PREs A, B and C in A2780 cells (*P* values=0.0193, 0.0115 and <0.0001, respectively, two-way ANOVA; Fig. 5d,e). Constructs containing the reference allele of PRE-A showed a silencing effect on the *ANKLE* promoter in both cell types with the risk allele further silencing the activity of the reference allele in A2780 cells (*P*=0.0049, two-way ANOVA). The reference allele of PRE-B had no effect on *ANKLE* promoter activity, while the risk allele significantly increased activity compared with the reference allele in A2780 cells (*P*=0.0034, two-way ANOVA). Constructs containing the reference allele of PRE-C significantly increased *ANKLE* promoter activity in both ovarian (*P*=0.0004, two-way ANOVA) and breast cell lines (*P*=0.0067, two-way ANOVA). However the risk allele showed a silencing effect on the reference allele in only Bre80 cells (*P*=0.0289, two-way ANOVA; Fig. 5d,e).

### Functional effects of rs56069439 deletion

**Figure 6:**
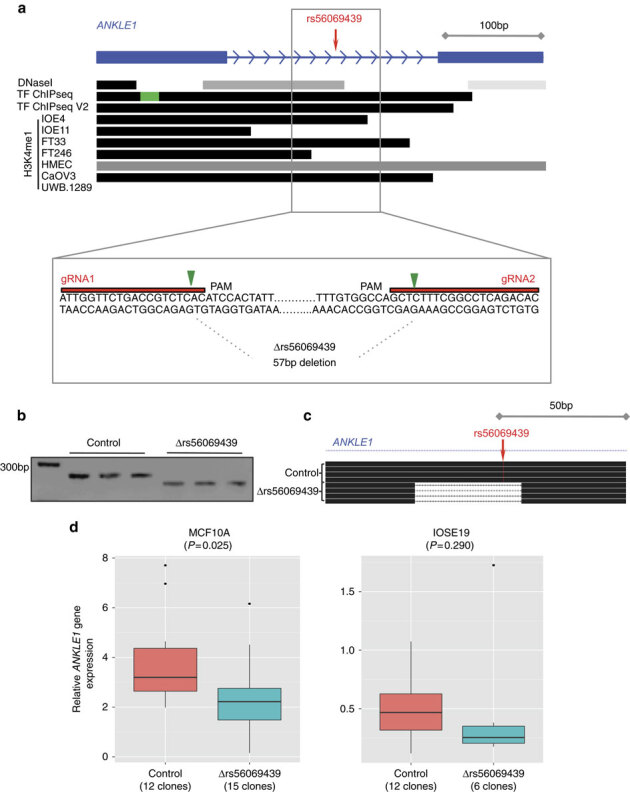
Effects of deletion of the putative enhancer containing the rs56069439 risk SNP in breast and ovarian epithelial cells. (**a**) Illustration of the 57 bp region in an intron of *ANKLE1* containing rs56069439; H3K4me1 marks overlapped rs56069439 in ovarian, fallopian and breast cells. Location of the two guide RNAs (gRNAs) used to create the stable Δrs56069439 deletion by CRISPR/Cas9 genome editing, cutting sites are indicated with the green arrow. PAM, protospacer adjacent motif. (**b**) PCR analysis of targeted region in representative MCF10A (breast) epithelial cell clones. Control clones were transfected with the vector backbone only. (**c**) Verification of deletions by Sanger sequencing, and alignment to the genome using BLAT. (**d**) Gene expression analysis using TaqMan probes showing downregulation of *ANKLE1* was associated with deletion of a region containing rs56069439.

Collectively, the data above suggested that rs56069439 may regulate the expression of *ANKLE1* and/or *ABHD8.* We used Clustered Regularly Interspaced Short Palindromic Repeats (CRISPR)/Cas9-mediated genome editing to delete a 57 bp region containing the regulatory region that includes rs56069439 in breast (MCF10A) and ovarian (IOSE19) epithelial cells (Fig. 6a). Analysis of multiple clones containing confirmed homozygous deletions (Fig. 6b,c) indicated a significant reduction in *ANKLE1* expression compared with parental cells (*P*=0.025, two-tailed paired *T*-test) and a trend towards reduced *ANKLE1* expression in IOSE19 cells (*P*=0.29, two-tailed paired *T*-test; Fig. 6d). Expression of *ABHD8* and *BABAM1* was unchanged following deletion of the region containing rs56069439.

### *In vitro* functional analysis of candidate genes

**Figure 7:**
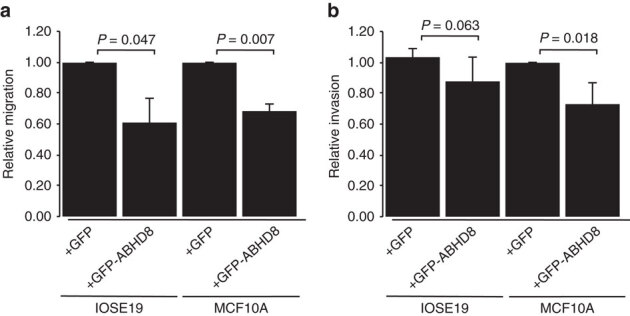
Phenotypic effects of overexpressing full length *ABHD8*GFP fusion transcript in normal breast and ovarian epithelial cells. (**a**) *ABHD8* overexpression induced a significant decrease in migration in both breast (MCF10A) and ovarian (IOSE19) cells; (**b**) *ABHD8* overexpression induced a significant decrease in invasion in breast epithelial cells and a similar trend of decreased invasion in ovarian epithelial cells.

We analysed the effects of perturbing *ABHD8, ANKLE1* and *BABAM1* expression in *in vitro* models of ‘normal’ breast (MCF10A) and ovarian (IOSE19 (ref. 32)) epithelial cells. For each gene, we overexpressed full length, green fluorescent protein-tagged constructs, because genes at 19p13 were frequently overexpressed in ovarian and BCs^9^ and because eQTL analyses indicated that risk alleles were associated with increased expression of *ABHD8* and *ANKLE1*. After confirming gene overexpression (Supplementary Fig. 3a) we evaluated cell growth, migration and invasion, and anchorage-independent growth (Fig. 7 and Supplementary Fig. 3b). Overexpression of *ABHD8* caused a significant reduction in cell migration (*P*=0.007 in MCF10A; *P*=0.047 in IOSE19, two-tailed paired *T*-test) and a decrease in invasion (*P*=0.018 in MCF10A; *P*=0.063 in IOSE19, two-tailed paired *T*-test; Fig. 7). *BABAM1* and *ANKLE1* overexpression had no effect on these cellular phenotypes for either cell type.

RNA sequencing was used to profile transcriptomic changes caused by overexpression of *ABHD8, ANKLE1* and *BABAM1* and pathway analyses performed using Ingenuity Pathway Analysis. We found no indication of significant changes in relevant pathways after overexpressing *BABAM1* in breast or ovarian epithelial cells. Cells overexpressing *ANKLE1* showed a significant enrichment for cancer-associated and cell growth/proliferation pathways in both breast (*P*=3.36 × 10^−6^) and ovarian (*P*=2.43 × 10^−27^) epithelial cells. Cells overexpressing *ABHD8* were enriched for expression changes in cancer related pathways (*P*<5.52 × 10^−8^) and fibrosis pathways (*P*<1.23 × 10^−2^, all right-tailed Fisher’s exact tests; Supplementary Tables 6-8).

## Discussion

Through fine-scale mapping of the 19p13.1 region we have found evidence of two independent regions of genetic association with BC and/or OC risk among women of European ancestry. The minor alleles of all candidate causal variants in Peak 1 conferred increased risks of ER-negative BC and serous OC and increased risks of both cancers for *BRCA1* mutation carriers. We were able to rule out associations with ER-positive BC and risks for other OC histotypes. There was weaker evidence that SNPs in Peak 2 were independently associated with BC risk among *BRCA1* mutation carriers only. When analyses in BCAC were restricted to triple-negative BC, the strength of association was greater and there was no evidence of association with ER-negative/HER2-positive BC. Thus, our results suggested that these variants are primarily associated with triple-negative BC, the predominant tumour subtype in *BRCA1* mutation carriers^33^. These results are in line with previous findings for the initial SNPs identified through GWAS^26^.

The increased sample size resulting from combining data from BCAC, OCAC and CIMBA for variants in Peak 1 have enabled us to restrict the likely functional variants at 19p13.1 to 13 SNPs. The 13 candidate causal risk SNPs in this region were the same for both BC and OC leading us to hypothesize that the underlying functional mechanisms are the same in both cancers and the overlap between these SNPs and functional elements provided multiple testable hypotheses, necessitating a range of different functional assays to evaluate their possible causality. Multiple assays were performed in breast and ovarian tissues and cell lines to establish if there is true evidence of pleiotropy. The candidate causal SNPs in Peak 1 clustered around two candidate genes, *ANKLE1* and *ABHD8*, neither of which have been previously implicated in BC or OC. Proximal to these SNPs is *BABAM1*, a gene involved in recruiting *BRCA1* to sites of DNA damage^34,35^ and therefore a compelling candidate gene at this locus. While gene regulation can be mediated across long genomic distances, the majority of interactions occur over a distance of 1 Mb) or less^36,37^. We, therefore, evaluated all candidate genes within a 1 Mb region centred on the Peak 1 risk SNPs for eQTL associations. We found significant eQTL associations for *ABHD8* in OCs and normal breast tissues, plus allele-specific expression of *ABHD8* in BCs, but no compelling evidence for any other gene at this locus. Nonetheless, the identification of *ABHD8* as the most likely target susceptibility gene must be treated with some caution as it is plausible that more distant *cis*-eQTL or even *trans*-eQTL associations exist for these risk SNPs. Unfortunately, the limited power of eQTL analysis based on the current sample size precluded us from performing genome-wide eQTL analysis to address these hypotheses.

The weight of our functional data, in particular the eQTL associations, indicates that *ABHD8* is a target of functional SNPs at this locus, and therefore a novel breast and OC susceptibility gene. 3C identified an interaction between a region containing four candidate causal SNPs and the *ABHD8* promoter in both breast and OC and normal epithelial cell lines. The luciferase assays of three PREs (including one encompassing rs56069439 in the interacting region) consistently showed that they acted as enhancers, and furthermore the risk-associated alleles of rs56069439, rs113299211, rs67397200, rs61494113, rs4808616 and rs55924783 (within PREs A-C) further increase *ABHD8* promoter activity in both breast and ovarian cells. These results were consistent with our eQTL studies and support the hypothesis that increased *ABHD8* expression is associated with an increased cancer risk. *ABHD8* is a poorly studied lipase^38^. The Achilles heel project identified *ABHD8* as a lineage-specific cancer cell vulnerability in OC cell lines^39^ and a recent study identified *ABHD8* as a potential OC susceptibility gene though its participation in a homeobox transcription factor-centred gene network associated with serous OC risk^40^. Overexpression of *ABHD8* led to significant reductions in the invasive and migratory potential of breast and ovarian cells and enriched for genes involved in cellular movement (IOSE19) and mTOR signalling (MCF10A), consistent with the observed changes in invasion and migration. The direction of the effect was opposite to what we might expect from the eQTL data, which might reflect different functions of ABHD8 in different contexts, similar to the observations for another BC susceptibility gene, *TOX3* (ref. 41). For example, under specific microenvironmental cues or in a tumour cell (rather than the normal cells used in these experiments) increased *ABHD8* may promote rather than inhibit migration and invasion.

Nonetheless, we cannot unequivocally exclude other genes as the targets of candidate causal variants at this locus, in particular *ANKLE1*. The close proximity of the candidate causal SNPs to the *ANKLE1* gene precluded 3C analysis; but in the luciferase assays, these same PREs and SNPs had variable, context-dependent effects on *ANKLE1* promoter activity. This raises the possibility that the SNPs were cooperatively acting to alter *ANKLE1* expression although it was difficult to predict the overall direction of their effects from this assay. We were able to rule out the SNP rs10419397 in the promoter of *ANKLE1* as a likely causal variant. The SNP rs111961716 in the 3′-UTR of *ANKLE1* was associated with allele-specific *ANKLE1* mRNA stability; but stable overexpression of *ANKLE1* had no influence on the phenotype of normal breast and ovarian epithelial cells even though pathway after overexpression of *ANKLE1* found a significant enrichment for cancer and cell death/proliferation associated pathways in both breast and ovarian epithelial cells. More recently, *ANKLE1* has been implicated in DNA damage responses, while other, better-characterized endonucleases (for example, ERCC1) are involved in nucleotide excision repair, which are important for the repair of bulky adducts^42^.

This study has highlighted the challenges in establishing causality for both candidate causal SNPs at common variant susceptibility loci and the susceptibility genes targets. The multitude of functional assays that can be used to test allele specific functional activity rarely provide unequivocal evidence of one SNP over another. Genome editing, which allows the creation of isogenic experimental models carrying the different alleles of candidate causal SNP, is emerging as a single assay approach that can evaluate the function of common variants. However, until now the technical challenges of genome editing have restricted its application to two non-coding risk SNPs identified by GWAS at susceptibility loci for prostate cancer and obesity, respectively^22,23^. It was beyond the scope of the current study to utilize genome editing to test all 13 candidate causal SNPs in Peak 1 at 19p13 in BC and OC and normal cell line models. Instead, we used CRISPR-Cas9 genome editing to evaluate the effects of a putative enhancer containing most plausible functional SNP (rs56069439) identified from 3C analysis and mapping of putative regulatory elements. This revealed strong functional evidence for a breast/ovarian epithelial cell enhancer, within an intron of *ANKLE1*. When this enhancer containing rs56069439 was deleted *ANKLE1* expression was significantly reduced, without any reduction in *BABAM1* or *ABHD8* expression. Further experiments using homology-directed repair will be required to determine if there is allele-specific activity of the rs56069439 SNP in regulating *ANKLE1* expression, and to determine whether shadow enhancers are employed to maintain *ABHD8* expression^43^.

In conclusion, we have performed detailed functional analysis of SNPs and candidate target genes at the 19p13 locus in breast and ovarian normal and cancer cells. *ABHD8* is the most likely target gene although we cannot rule out a role for *ANKLE1* in the development of breast and OC or the possibility that both genes, acting independently or in synergy may be functional targets of candidate causal SNPs. Using a combination of genetic fine mapping, and a spectrum of *in silico* and functional assays, seven of thirteen showed evidence of functionality.

These data suggest that the underlying functional mechanism(s) at the 19p13 locus may be mediated by many SNPs rather than by a single causal allele. This hypothesis is supported by studies showing tissue-specific enrichment of correlated risk-associated SNPs at susceptibility loci within regulatory biofeatures, including enhancers and transcription factor binding sites^19,20^. Such enrichments would not be detected if a single causal SNP at a locus was driving disease development. Taken together these data suggest that common molecular mechanisms are likely to underlie this pleiotropic risk locus.

## Methods

### Study populations

All specimens used in this study were collected with informed consent and under the approval of local Institutional Review Boards. We used epidemiological and genotype data from studies participating in the BCAC^44^, the OCAC^12^ and the CIMBA^45^ that have been genotyped using the iCOGS array that included ∼200,000 SNPs.

### BC association consortium

Data were available from 52 BC case-control studies, 41 studies of European ancestry, 9 studies of Asian ancestry and 2 studies of African-American ancestry. Details of all studies, the genotyping process and the quality control process have been described elsewhere^6,44^, standard sample and genotyping QC criteria were applied. After the quality control process, data on 46,451 cases and 42,599 controls of European ancestry, 6,269 cases and 6,624 controls of Asian ancestry and 1,117 cases and 932 controls of African-American ancestry were available for analysis. Data on the BC ER status were available for 34,509 cases of European ancestry, 7,435 (22%) of whom had ER-negative tumours.

### OC association consortium

Data were available from 41 case-control studies of EOC from OCAC that were genotyped using the iCOGS array^12^. In addition to the OCAC iCOGS data, genotype data were available for stage 1 of three population-based OC genome-wide association studies. The final data set comprised genotype data for 11,069 cases and 21,722 controls from COGS (‘OCAC-iCOGS’), 2,165 cases and 2,564 controls from a GWAS from North America (‘US GWAS’)^46^, 1,762 cases and 6,118 controls from a UK-based GWAS (‘UK GWAS’)^7^, and 441 cases and 441 controls from the Mayo Clinic. All subjects included in this analysis provided written informed consent as well as data and blood samples under ethically approved protocols. Overall, 43 studies from 11 countries provided data on 15,437 women diagnosed with invasive EOC, 9,627 of whom were diagnosed with serous EOC and 30,845 controls from the general population.

### Consortium of investigators of modifiers of BRCA1/2

Data on *BRCA1* mutation carriers were obtained through CIMBA. Eligibility in CIMBA is restricted to females 18 years or older with pathogenic mutations in *BRCA1* or *BRCA2*. The majority of the participants were sampled through cancer genetics clinics^47^, including some related participants. Fifty-one studies from 25 countries contributed data on *BRCA1* mutation carriers who were genotyped using the iCOGS array^45^. After quality control of the phenotypes and genotypes, data were available on 15,252 *BRCA1* mutation carriers of whom 7,455 had been diagnosed with BC, 2,639 with ER-negative BC and 1,724 with OC, all of European ancestry. Analyses in *BRCA1* mutation carriers focused on assessing associations with BC risk, following the evidence from the original GWAS in *BRCA1* mutation carriers^48^.

URLs: 1000 Genomes Project, http://www.1000genomes.org/; BCAC, http://ccge.medschl.cam.ac.uk/consortia/bcac/index.html; CIMBA, http://ccge.medschl.cam.ac.uk/consortia/cimba/index.html; COGS, http://www.cogseu.org/; iCOGS, http://ccge.medschl.cam.ac.uk/research/consortia/ icogs/; SNAP

https://www.broadinstitute.org/mpg/snap/; TCGA, https://tcga-data.nci.nih.gov; CGHub, https://cghub.ucsc.edu/

### iCOGS SNP selection for fine mapping and imputation

The fine mapping region was defined as Chromosome 19 positions: 17,130,000–17,550,000 (NCBI build 37). To identify the set of variants potentially responsible for the original GWAS reports, we considered all variants with minor allele frequencies of >0.02 from the 1,000 Genomes Project (March 2010 version) and selected all SNPs correlated (*r*^2^>0.1) with either of the two SNPs that had been identified through the *BRCA1* and EOC GWAS studies (rs8170 and rs2363956)^12,45^, plus an additional set of SNPs that tagged all remaining SNPs in the region with *r*^2^>0.9. A total of 438 SNPs that were included on iCOGS in the 19p13 region passed QC and were available for the analyses. Data on these SNPs were used to impute the genotypes of all known variants from the 1,000 genomes project (V3, April 2012 release49) using the IMPUTE (version 2) software. After excluding SNPs with MAF<0.001 and SNPs with imputation *r*^2^ accuracy score of ≤0.3, there were 2,269 imputed SNPs in BCAC, 2,565 in OCAC and 2,311 in *BRCA1* mutation.

### BCAC and OCAC association analysis and logistic regression

To evaluate the association of each SNP with breast and EOC risk in BCAC and OCAC we used a Wald test statistic based on logistic regression, by estimating the per-allele OR and its s.e. Analyses restricted to specific tumour subtypes (ER-negative BC or high-grade serous EOC) were assessed separately using all available controls. All analyses were adjusted for principal components, described in more detail elsewhere^12,44^. Conditional logistic regression was used to assess the evidence that there are multiple independent association signals in the region, by evaluating the associations of genetic variants in the region while adjusting for the SNP with the smallest *P* value. We considered only SNPs with *P* values of association of <10^−3^ and MAF>0.1% and the most parsimonious model was identified using step-wise forward logistic regression and a threshold of *P*<10^−4^ for retaining SNPs in the model.

### CIMBA retrospective cohort analysis

All associations between genotypes and BC risk in *BRCA1* mutation carriers were evaluated using a 1 *df* per allele trend-test (*P*-trend), based on modelling the retrospective likelihood of the observed genotypes conditional on BC phenotypes^49^. To allow for the non-independence among related individuals, an adjusted test statistic was used which took into account the correlation in genotypes^48^. Per allele HR estimates were obtained by maximizing the retrospective likelihood. All analyses were stratified by country of residence. To identify the most parsimonious model that includes multiple SNPs, forward-selection Cox-regression analysis was performed, using the same *P* value thresholds as in the BCAC and OCAC analysis. This approach provides valid tests of association, although the parameter estimates can be biased^49,50^. Parameter estimates for the most parsimonious model were obtained using the retrospective likelihood approach.

### Meta-analysis

It is well established that the majority of BCs in *BRCA1* mutation carriers are ER-negative^51,52^. To increase the statistical power for identifying the most likely causal variants, we also performed a meta-analysis of the associations of BC risk for *BRCA1* mutation carriers and ER-negative BC in the general population (in BCAC) for both genotyped and imputed SNPs. We used an inverse variance approach assuming mixed effects, by combining the logarithm of the per-allele HR for the association with BC risk for *BRCA1* mutation carriers and the logarithm of the OR estimate for the association with ER-negative BC in BCAC.

### eQTL and allele-specific expression analyses

Germline genotype data were obtained from the Affymetrix SNP 6.0 (METABRIC) and Illumina 1M-Duo (TCGA HGSOC). No SNPs from Peak 1 and 2 were present on the Affymetrix platform so these genotypes were imputed into the 1000 Genomes European reference panel (March 2012, version 3) using IMPUTE version 2 (ref. 53). All analyses were restricted to patients of >90% European ancestry as per LAMP estimates^54^ and SNPs with info score >0.3. For METABRIC, gene expression data consisted of probe-level measurements from the Illumina HT-12 v3 microarray platform for a total of 135 samples obtained from normal breast tissue adjacent to tumour and 59 samples obtained from ER-negative breast tumours were analysed. For TCGA HGSOC, gene expression data consisted of measurements from the Agilent 244 K microarray for 340 HGSOC tumours downloaded from the cBioportal. Only genes and probes <1 Mb from the top Peak 1 SNP were analysed. Tumour gene expression data was first adjusted for copy number (TCGA and METBRIC, Affymetrix SNP 6.0 calls) and methylation (TCGA only, Illumina 27 K beta values) using the method of Li *et al*^31^. Expression QTL analysis was conducted by linear regression with genotypes as predictors, as implemented in the R package Matrix eQTL^55^.

Sixty early passage primary normal OSECs and fallopian tube epithelial cells were collected and cultured as previously described^27,56^. Briefly, OSECs were harvested from ovaries using a sterile cytobrush and cultured in Medium 199 and MCDB105, mixed in a 1:1 ratio and supplemented with 15% fetal bovine serum (FBS, Hyclone), 10 ng ml^−1^ epidermal growth factor, 0.5 mg ml^−1^ hydrocortisone, 5 mg ml^−1^ insulin (all Sigma, St Louis, MO, USA) and 34 mg protein per ml bovine pituitary extract (Life Technologies). Fresh fallopian specimens were subjected to 48–72 h Pronase (Roche) and DNase I digests to release the epithelial cells. Epithelial cells were pelleted and cultured on collagen in DMEM/F12 supplemented with 10% FBS (Seradigm). RNA was isolated from cell cultures harvested at ∼80% confluency using the QIAgen miRNAeasy kit with on-column DNase 1 digestion. 500 ng of RNA was reverse transcribed using SuperScript III First-Strand Synthesis System (Invitrogen). The cDNA was diluted to 10 ng μl^−1^ and 12.5 ng was used in target specific amplification before real-time PCR using TaqMan PreAmp Master Mix Kit (Applied Biosystems) following Fluidigm’s Specific Target Amplification Protocol. 1.25 μl of the 25 μl pre-amplified cDNA was added to each chip. Each sample was run in triplicate and each experiment included no template controls and no template controls from the cDNA reactions. 96.96 Dynamic Array Integrated Fluidic Circuits (Fluidigm) were loaded with 96 pre-amplified cDNA samples and 96 TaqMan gene expression probes (Applied Biosystems) using the BioMark HD System (Fluidigm). Expression levels for each gene were normalized to the average expression of control genes (*GAPDH* and *ACTB*). Relative expression levels were calculated using the ΔΔCt method. Correlations between genotype and gene expression were calculated in R 2.14.1. Genotype specific gene expression was compared using the Jonckheere–Terpstra test. Genes with significant eQTL results were validated by individual Taqman (Applied Biosystems, Warrington UK) reactions run on ABI 7900HT Sequence Detection System equipment and analysed with SDS software according to the manufacturer’s instructions. Normal cell line DNAs were analysed on iCOGS arrays to obtain genotype information. We analysed all protein-coding genes within a 1 Mb region of the risk association. The method for allele specific expression analysis has been described previously^31^.

### Breast and ovarian normal and cancer cell lines

Breast and OC cell lines MCF7 (ER+, breast; ATCC #HTB-22) and A2780 (ER+, ovarian; kindly provided by Thomas Hamilton, NCI, Maryland) were grown in RPMI medium with 10% FBS and antibiotics. The normal breast epithelial cell lines Bre-80 (kindly provided by Roger Reddel, CMRI, Sydney) and MCF10A (ATCC #CRL-10317) were grown in DMEM/F12 medium with 5% horse serum, 10 mg ml^−1^ insulin, 0.5 mg ml^−1^ hydrocortisone, 20 ng ml^−1^ epidermal growth factor, 100 ng ml^−1^ cholera toxin and antibiotics. The phenotypically normal TERT immortalized ovarian epithelial cell lines IOSE11 and IOSE19 (ref. 32) were grown in NOSE-CM. All cell lines were maintained under standard conditions, were routinely tested for *Mycoplasma* and were profiled with short tandem repeats to confirm their identity.

### Functional annotation of risk SNPs

FAIRE-seq and ChIP-seq for H3K27ac and H3K4me1 marks in normal ovarian (IOSE4, IOSE11) and fallopian epithelial cell lines (FT33, FT246) and OC cell lines (CaOV3, UWB1.289) were generated in-house using standard protocols and have been previously described^19,27^. Epigenetic marks in HMECs were downloaded from ENCODE (genome.ucsc.edu).

### Chromosome conformation capture

3C libraries were generated using *Nco*I as described previously^14^. To quantify interactions by real-time quantitative PCR (qPCR) was performed using primers listed in Supplementary Table 9. All qPCRs were performed on a RotorGene 6,000 using MyTaq HS DNA polymerase with the addition of 5 mM of Syto9, annealing temperature of 66 °C and extension of 30 s. Each experiments was performed three times in duplicate. The BAC clone (CTD-2278I10) covering the 19p13 region was used to normalize for PCR efficiency and a by reference region within *GAPDH* used to calculate relative interaction frequencies. All qPCR products were resolved on 2% agarose gels, gel purified and sequenced to verify the 3C product.

### RNA stability assays

For each genotype (two homozygotes and the heterozygote) two early passage primary normal ovarian epithelial cell lines were incubated with actinomycin D for 20 h. RNA was extracted using the QIAgen RNeasy extraction kit and reverse transcribed using MMLV RT enzyme and random hexamers (Promega). Quantitative PCR was performed using TaqMan gene expression probes for *ABHD8* (Hs00225984_m1) and *ANKLE1* (Hs01094673_g1). Signal for each gene of interest was normalized to signal for *ACTB* (Hs01060665_g1) and *GAPDH* (Hs02758991_g1) and relative gene expression calculated using the ΔΔCt method, relative to untreated cells. 18s rRNA (Hs99999901_s1) and *MYC* (Hs00153408_m1) mRNA levels were included as internal controls.

### Promoter and allele specific enhancer assays

A 1119, bp fragment containing the *ABHD8* promoter was cloned into the pGL3 basic luciferase reporter. Reference and risk associated *ANKLE1* promoter fragments were synthesized by GenScript and cloned into pGL3 basic. We generated PCR fragments corresponding to PRE A and PRE B and had PRE C haplotype fragments synthesized by GenScript and these were also sub-cloned into *ABHD8* and *ANKLE1* promoter constructs. PCR primers are listed in Supplementary Table 10. Bre80 and A2780 cells were transiently transfected with equimolar amounts of luciferase reporter constructs using *Renilla* luciferase as an internal control reporter. Luciferase was measured 24 h after transfection using Dual-Glo Luciferase (Promega). To correct for any differences in transfection efficiency or cell lysate preparation, *Firefly* luciferase activity was normalized to *Renilla* luciferase, and the activity of each construct was measured relative to the promoter alone construct, which had a defined activity of 1. Association was assessed by log transforming the data and performing two-way ANOVA, followed by Dunnett’s multiple comparisons test; for ease of interpretation, values were back transformed to the original scale for the graphs.

### Genome editing

Guide RNAs targeting the region flanking rs56069439 (5′-GTGAGACGGTCAGAACCAAT-3′ and 5′-GTGTCTGAGGCCGAAAGAGC-3′) were designed using the CRISPR design tool from the Zhang lab (www.crispr.mit.edu)^57^. The gRNAs were cloned into the lentiCRISPR (Addgene Plasmid 49535) vector by using the *BsmBI* restriction enzyme site and lentiviral supernatants made by cotransfection of HEK293T cells. IOSE19 and MCF10A cells were transduced with viral supernatants and infected cells selected using 400 ng ml^−1^ and 500 ng ml^−1^ puromycin (Sigma Aldrich) respectively. Selected cells were sorted into single cells using flow cytometry and expanded *in vitro.* Screening for clones containing the deletion was performed using the following primers: Forwards: 5′-CCCTGACATCCAGGGTCTTC-3′ and Reverse: 5′-AGTCCAGCGTCTCATCGGTA-3′. For sequence verification of the deletion the following primers were used: Forwards: 5′-TTCTGGACCAGTCCCTGACA-3′ and Reverse: 5′-CAGCGTCTCATCGGTAGGTC-3′. RNA was isolated from positive clones using the Zymo Quick-RNA kit and reverse transcribed using Superscript III (Life Technologies). Real time gene expression analysis was performed using TaqMan probes, as described above.

### *In vitro* analysis of candidate genes

The three candidate genes were overexpressed as green fluorescent protein fusion proteins. The *BABAM1* overexpression construct was a kind gift from Dr S Elledge^58^. *ANKLE1* and *ABHD8* contructs were purchased from Genecopoeia. Virus was made in-house by cotransfection of HEK293Ts and used to transduce MCF10A and IOSE19 cells. Positive cells were selected using 400 ng ml^−1^ (for IOSE19 cells) or 500 ng ml^−1^ (for MCF10A cells) puromycin. Anchorage dependent and independent growth assays were performed as previously described^32,59^. For invasion and migration assays Millipore luminescent transwell assays (24 well plate format) were used, following the manufacturer’s protocol.

### Data availability

The relevant SNP genotype data underpinning these analyses can be accessed by applying to the OCAC, BCAC and CIMBA consortia (see URLs). EQTL data are available in supplementary information. All other data are available on request.

## Additional information

**How to cite this article:** Lawrenson, K. *et al*. Functional mechanisms underlying pleiotropic risk alleles at the 19p13.1 breast-ovarian cancer susceptibility locus. *Nat. Commun.* 7:12675 doi: 10.1038/ncomms12675 (2016).

## Supplementary information

Supplementary Information Supplementary Figures 1-4, Supplementary Tables 1-10 and Supplementary References. (PDF 1359 kb)

Supplementary Data 1 EQTL analyses in normal breast tissues from the METABRIC study. P-values from linear regression analyses. (XLSX 49 kb)

Supplementary Data 2 Expression QTL analyses in normal primary ovarian and fallopian tube secretory epithelial cells, using Fluidigm gene expression chips and TaqMan probes. Positive results were verified using the 7900HT fast real-time PCR system (Life Technologies). # The positive result for MYO9B was not reproducible but † the ANKLE1 association was verified (P=0.002). (XLSX 41 kb)

Supplementary Data 3 Significant allele specific expression in breast cancer samples from TCGA (Peak 1) (P<0.05, F-tests). No associations were observed for any Peak 2 SNPs. (XLSX 38 kb)
