# Author Correction: Functional mechanisms underlying pleiotropic risk alleles at the 19p13.1 breast–ovarian cancer susceptibility locus

**DOI:** 10.1038/s41467-025-63507-x

**Published:** 2025-09-08

**Authors:** Kate Lawrenson, Siddhartha Kar, Karen McCue, Karoline Kuchenbaeker, Kyriaki Michailidou, Jonathan Tyrer, Jonathan Beesley, Susan J. Ramus, Qiyuan Li, Melissa K. Delgado, Janet M. Lee, Kristiina Aittomäki, Irene L. Andrulis, Hoda Anton-Culver, Volker Arndt, Banu K. Arun, Brita Arver, Elisa V. Bandera, Monica Barile, Rosa B. Barkardottir, Daniel Barrowdale, Matthias W. Beckmann, Javier Benitez, Andrew Berchuck, Maria Bisogna, Line Bjorge, Carl Blomqvist, William Blot, Natalia Bogdanova, Anders Bojesen, Stig E. Bojesen, Manjeet K. Bolla, Bernardo Bonanni, Anne-Lise Børresen-Dale, Hiltrud Brauch, Paul Brennan, Hermann Brenner, Fiona Bruinsma, Joan Brunet, Shaik Ahmad Buhari, Barbara Burwinkel, Ralf Butzow, Saundra S. Buys, Qiuyin Cai, Trinidad Caldes, Ian Campbell, Rikki Cannioto, Jenny Chang-Claude, Jocelyne Chiquette, Ji-Yeob Choi, Kathleen B. M. Claes, GEMO Study Collaborators, Marie-Agnès Collonge-Rame, Alexandre Damette, Emmanuelle Barouk-Simonet, Françoise Bonnet, Virginie Bubien, Nicolas Sevenet, Michel Longy, Pascaline Berthet, Dominique Vaur, Laurent Castera, Sandra Fert Ferrer, Yves-Jean Bignon, Nancy Uhrhammer, Fanny Coron, Laurence Faivre, Amandine Baurand, Caroline Jacquot, Geoffrey Bertolone, Sarab Lizard, Dominique Leroux, Hélène Dreyfus, Christine Rebischung, Magalie Peysselon, Jean-Philippe Peyrat, Joëlle Fournier, Françoise Révillion, Claude Adenis, Laurence Vénat-Bouvet, Mélanie Léone, Nadia Boutry-Kryza, Alain Calender, Sophie Giraud, Carole Verny-Pierre, Christine Lasset, Valérie Bonadona, Laure Barjhoux, Hagay Sobol, Violaine Bourdon, Tetsuro Noguchi, Audrey Remenieras, Isabelle Coupier, Pascal Pujol, Johanna Sokolowska, Myriam Bronner, Capucine Delnatte, Stéphane Bézieau, Véronique Mari, Marion Gauthier-Villars, Bruno Buecher, Etienne Rouleau, Lisa Golmard, Virginie Moncoutier, Muriel Belotti, Antoine de Pauw, Camille Elan, Emmanuelle Fourme, Anne-Marie Birot, Claire Saule, Maïté Laurent, Claude Houdayer, Fabienne Lesueur, Noura Mebirouk, Florence Coulet, Chrystelle Colas, Florent Soubrier, Mathilde Warcoin, Fabienne Prieur, Marine Lebrun, Caroline Kientz, Danièle Muller, Jean-Pierre Fricker, Christine Toulas, Rosine Guimbaud, Laurence Gladieff, Viviane Feillel, Isabelle Mortemousque, Brigitte Bressac-de-Paillerets, Olivier Caron, Marine Guillaud-Bataille, Linda S. Cook, Angela Cox, Daniel W. Cramer, Simon S. Cross, Cezary Cybulski, Kamila Czene, Mary B. Daly, Francesca Damiola, Agnieszka Dansonka-Mieszkowska, Hatef Darabi, Joe Dennis, Peter Devilee, Orland Diez, Jennifer A. Doherty, Susan M. Domchek, Cecilia M. Dorfling, Thilo Dörk, Martine Dumont, Hans Ehrencrona, Bent Ejlertsen, Steve Ellis, EMBRACE, Helen Gregory, Zosia Miedzybrodzka, Patrick J. Morrison, Alan Donaldson, Mark T. Rogers, M. John Kennedy, Mary E. Porteous, Angela Brady, Julian Barwell, Claire Foo, Fiona Lalloo, Lucy E. Side, Jacqueline Eason, Alex Henderson, Lisa Walker, Jackie Cook, Katie Snape, Alex Murray, Emma McCann, Christoph Engel, Eunjung Lee, D. Gareth Evans, Peter A. Fasching, Lidia Feliubadalo, Jonine Figueroa, Dieter Flesch-Janys, Olivia Fletcher, Henrik Flyger, Lenka Foretova, Florentia Fostira, William D. Foulkes, Brooke L. Fridley, Eitan Friedman, Debra Frost, Gaetana Gambino, Patricia A. Ganz, Judy Garber, Montserrat García-Closas, Aleksandra Gentry-Maharaj, Maya Ghoussaini, Graham G. Giles, Rosalind Glasspool, Andrew K. Godwin, Mark S. Goldberg, David E. Goldgar, Anna González-Neira, Ellen L. Goode, Marc T. Goodman, Mark H. Greene, Jacek Gronwald, Pascal Guénel, Christopher A. Haiman, Per Hall, Emily Hallberg, Ute Hamann, Thomas V. O. Hansen, Patricia A. Harrington, Mikael Hartman, Norhashimah Hassan, Sue Healey, The Hereditary Breast and Ovarian Cancer Research Group Netherlands (HEBON), M. A. Rookus, F. E. van Leeuwen, L. E. van der Kolk, M. K. Schmidt, N. S. Russell, J. L. de Lange, R. Wijnands, J. M. Collée, M. J. Hooning, C. Seynaeve, C. H. M. van Deurzen, I. M. Obdeijn, C. J. van Asperen, R. A. E. M. Tollenaar, T. C. T. E. F. van Cronenburg, C. M. Kets, M. G. E. M. Ausems, C. C. van der Pol, T. A. M. van Os, Q. Waisfisz, H. E. J. Meijers-Heijboer, E. B. Gómez-Garcia, J. C. Oosterwijk, M. J. Mourits, G. H. de Bock, H. F. Vasen, S. Siesling, J. Verloop, L. I. H. Overbeek, Florian Heitz, Josef Herzog, Estrid Høgdall, Claus K. Høgdall, Frans B. L. Hogervorst, Antoinette Hollestelle, John L. Hopper, Peter J. Hulick, Tomasz Huzarski, Evgeny N. Imyanitov, KConFab Investigators, Stephen Fox, Judy Kirk, Geoff Lindeman, Melanie Price, Australian Ovarian Cancer Study Group, David Bowtell, Anna deFazio, Penny Webb, Claudine Isaacs, Hidemi Ito, Anna Jakubowska, Ramunas Janavicius, Allan Jensen, Esther M. John, Nichola Johnson, Maria Kabisch, Daehee Kang, Miroslav Kapuscinski, Beth Y. Karlan, Sofia Khan, Lambertus A. Kiemeney, Susanne Kruger Kjaer, Julia A. Knight, Irene Konstantopoulou, Veli-Matti Kosma, Vessela Kristensen, Jolanta Kupryjanczyk, Ava Kwong, Miguel de la Hoya, Yael Laitman, Diether Lambrechts, Nhu Le, Kim De Leeneer, Jenny Lester, Douglas A. Levine, Jingmei Li, Annika Lindblom, Jirong Long, Artitaya Lophatananon, Jennifer T. Loud, Karen Lu, Jan Lubinski, Arto Mannermaa, Siranoush Manoukian, Loic Le Marchand, Sara Margolin, Frederik Marme, Leon F. A. G. Massuger, Keitaro Matsuo, Sylvie Mazoyer, Lesley McGuffog, Catriona McLean, Iain McNeish, Alfons Meindl, Usha Menon, Arjen R. Mensenkamp, Roger L. Milne, Marco Montagna, Kirsten B. Moysich, Kenneth Muir, Anna Marie Mulligan, Katherine L. Nathanson, Roberta B. Ness, Susan L. Neuhausen, Heli Nevanlinna, Silje Nord, Robert L. Nussbaum, Kunle Odunsi, Kenneth Offit, Edith Olah, Olufunmilayo I. Olopade, Janet E. Olson, Curtis Olswold, David O’Malley, Irene Orlow, Nick Orr, Ana Osorio, Sue Kyung Park, Celeste L. Pearce, Tanja Pejovic, Paolo Peterlongo, Georg Pfeiler, Catherine M. Phelan, Elizabeth M. Poole, Katri Pylkäs, Paolo Radice, Johanna Rantala, Muhammad Usman Rashid, Gad Rennert, Valerie Rhenius, Kerstin Rhiem, Harvey A. Risch, Gus Rodriguez, Mary Anne Rossing, Anja Rudolph, Helga B. Salvesen, Suleeporn Sangrajrang, Elinor J. Sawyer, Joellen M. Schildkraut, Marjanka K. Schmidt, Rita K. Schmutzler, Thomas A. Sellers, Caroline Seynaeve, Mitul Shah, Chen-Yang Shen, Xiao-Ou Shu, Weiva Sieh, Christian F. Singer, Olga M. Sinilnikova, Susan Slager, Honglin Song, Penny Soucy, Melissa C. Southey, Marie Stenmark-Askmalm, Dominique Stoppa-Lyonnet, Christian Sutter, Anthony Swerdlow, Sandrine Tchatchou, Manuel R. Teixeira, Soo H. Teo, Kathryn L. Terry, Mary Beth Terry, Mads Thomassen, Maria Grazia Tibiletti, Laima Tihomirova, Silvia Tognazzo, Amanda Ewart Toland, Ian Tomlinson, Diana Torres, Thérèse Truong, Chiu-chen Tseng, Nadine Tung, Shelley S. Tworoger, Celine Vachon, Ans M. W. van den Ouweland, Helena C. van Doorn, Elizabeth J. van Rensburg, Laura J. Van’t Veer, Adriaan Vanderstichele, Ignace Vergote, Joseph Vijai, Qin Wang, Shan Wang-Gohrke, Jeffrey N. Weitzel, Nicolas Wentzensen, Alice S. Whittemore, Hans Wildiers, Robert Winqvist, Anna H. Wu, Drakoulis Yannoukakos, Sook-Yee Yoon, Jyh-Cherng Yu, Wei Zheng, Ying Zheng, Kum Kum Khanna, Jacques Simard, Alvaro N. Monteiro, Juliet D. French, Fergus J. Couch, Matthew L. Freedman, Douglas F. Easton, Alison M. Dunning, Paul D. Pharoah, Stacey L. Edwards, Georgia Chenevix-Trench, Antonis C. Antoniou, Simon A. Gayther

**Affiliations:** 1 Department of Preventive Medicine, Keck School of Medicine, University of Southern California Norris Comprehensive Cancer Center, Los Angeles, 90033 California USA; 2 Department of Oncology, Centre for Cancer Genetic Epidemiology, University of Cambridge, Cambridge, CB1 8RN UK; 3 QIMR Berghofer Medical Research Institute, Brisbane, 4029 Queensland Australia; 4 Department of Public Health and Primary Care, Centre for Cancer Genetic Epidemiology, University of Cambridge, Cambridge, CB1 8RN UK; 5 Medical College, Xiamen University, Xiamen, 361102 China; 6 Department of Medical Oncology, The Center for Functional Cancer Epigenetics, Dana-Farber Cancer Institute, Boston, 02215 Massachusetts USA; 7 Department of Clinical Genetics, Helsinki University Hospital, University of Helsinki, Helsinki, 00029 HUS Finland; 8 Lunenfeld-Tanenbaum Research Institute of Mount Sinai Hospital, Toronto, M5G 1X5 Ontario Canada; 9 Department of Molecular Genetics, University of Toronto, Toronto, M5S 1A8 Ontario Canada; 10 Department of Epidemiology, Genetic Epidemiology Research Institute, School of Medicine, University of California Irvine, Irvine, 92697 California USA; 11 Division of Clinical Epidemiology and Aging Research, German Cancer Research Center (DKFZ), Heidelberg, 69120 Germany; 12 University of Texas MD Anderson Cancer Center, Houston, 77030 Texas USA; 13 Department of Oncology, Karolinska University Hospital, Stockholm, 171 77 Sweden; 14 Cancer Prevention and Control, Rutgers Cancer Institute of New Jersey, New Brunswick, 08903 New Jersey USA; 15 Division of Cancer Prevention and Genetics, Istituto Europeo di Oncologia, Milan, 20141 Italy; 16 Department of Pathology, Landspitali University Hospital and BMC (Biomedical Centre), Faculty of Medicine, University of Iceland, Reykjavik, 600169-2039 Iceland; 17 Department of Gynecology and Obstetrics, University Hospital Erlangen, Friedrich-Alexander-University Erlangen-Nuremberg, Comprehensive Cancer Center Erlangen-EMN, Erlangen, 91054 Germany; 18 Human Cancer Genetics Program, Spanish National Cancer Research Centre, Madrid, E-28029 Spain; 19 Centro de Investigación en Red de Enfermedades Raras, Valencia, 28029 Spain; 20 Department of Obstetrics and Gynecology, Duke University Medical Center, Durham, 27710 North Carolina USA; 21 Department of Surgery, Gynecology Service, Memorial Sloan-Kettering Cancer Center, New York, 10065 USA; 22 Department of Gynecology and Obstetrics, Haukeland University Hospital, Bergen, 5021 Norway; 23 Department of Clinical Science, Centre for Cancer Biomarkers, University of Bergen, Bergen, N-5020 Norway; 24 Department of Oncology, Helsinki University Hospital, University of Helsinki, Helsinki, FIN-00029 Finland; 25 Division of Epidemiology, Department of Medicine, Vanderbilt-Ingram Cancer Center, Vanderbilt University School of Medicine, Nashville, 37203 Tennessee USA; 26 International Epidemiology Institute, Rockville, 20850 Maryland USA; 27 Gynaecology Research Unit, Hannover Medical School, Hannover, D-30625 Germany; 28 Department of Clinical Genetics, Vejle Hospital, Vejle, 7100 Denmark; 29 Faculty of Health and Medical Sciences, University of Copenhagen, Copenhagen, 2200 Denmark; 30 Department of Clinical Biochemistry, Herlev Hospital, Copenhagen University Hospital, Herlev, 2730 Denmark; 31 Copenhagen General Population Study, Herlev Hospital, Copenhagen University Hospital, Herlev, 2730 Denmark; 32 Department of Genetics, Institute for Cancer Research, Oslo University Hospital Radiumhospitalet, Oslo, N-0310 Norway; 33 K.G. Jebsen Center for Breast Cancer Research, Institute of Clinical Medicine, Faculty of Medicine, University of Oslo, Oslo, N-0310 Norway; 34 Dr Margarete Fischer-Bosch-Institute of Clinical Pharmacology, Stuttgart, D-70376 Germany; 35 University of Tübingen, Tübingen, 72074 Germany; 36 German Cancer Consortium (DKTK), German Cancer Research Center (DKFZ), Heidelberg, 69120 Germany; 37 International Agency for Research on Cancer, Lyon, 69008 France; 38 Division of Preventive Oncology, German Cancer Research Center (DKFZ), Heidelberg, 69121 Germany; 39 Cancer Epidemiology Centre, Cancer Council Victoria, Melbourne, 3004 Victoria Australia; 40 Genetic Counseling Unit, Hereditary Cancer Program, IDIBGI (Institut d’Investigació Biomèdica de Girona), Catalan Institute of Oncology, Girona, 08908 Spain; 41 Department of Surgery, National University Health System, Singapore, 119077 Singapore; 42 Molecular Epidemiology Group, German Cancer Research Center (DKFZ), Heidelberg, 69120 Germany; 43 Department of Obstetrics and Gynecology, University of Heidelberg, Heidelberg, 69120 Germany; 44 Department of Obstetrics and Gynecology, University of Helsinki and Helsinki University Central Hospital, Helsinki, 00029 HUS Finland; 45 Department of Pathology, Helsinki University Central Hospital, Helsinki, 00029 Finland; 46 Department of Medicine, Huntsman Cancer Institute, University of Utah School of Medicine, Salt Lake City, 84112 Utah USA; 47 Molecular Oncology Laboratory, Hospital Clinico San Carlos, IdISSC (El Instituto de Investigación Sanitaria del Hospital Clínico San Carlos), Madrid, 28040 Spain; 48 Cancer Genetics Laboratory, Peter MacCallum Cancer Centre, Melbourne, 3002 Victoria Australia; 49 Division of Cancer Prevention and Population Sciences, Cancer Pathology & Prevention, Roswell Park Cancer Institute, Elm and Carlton Streets, Buffalo, 14263 New York USA; 50 Division of Cancer Epidemiology, German Cancer Research Center (DKFZ), Heidelberg, 69121 Germany; 51 University Cancer Center Hamburg (UCCH), University Medical Center Hamburg-Eppendorf, Hamburg, 20246 Germany; 52 Unité de recherche en santé des populations, Centre des maladies du sein Deschênes-Fabia, Centre de recherche FRSQ du Centre hospitalier affilié universitaire de Québec, Québec City, G1J 1Z4 Québec Canada; 53 Cancer Research Institute, Seoul National University, Seoul, 08826 Korea; 54 Department of Biomedical Sciences, Seoul National University College of Medicine, Seoul, 03080 Korea; 55 Center for Medical Genetics, Ghent University, Ghent, 9000 Belgium; 56 Division of Epidemiology and Biostatistics, Department of Internal Medicine, University of New Mexico, Albuquerque, 87131 New Mexico USA; 57 Department of Oncology, Sheffield Cancer Research, University of Sheffield, Sheffield, S10 2TN UK; 58 Harvard HT Chan School of Public Health, Boston, 02115 Massachusetts USA; 59 Obstetrics and Gynecology Epidemiology Center, Brigham and Women’s Hospital and Harvard Medical School, Boston, 02115 Massachusetts USA; 60 Department of Neuroscience, Academic Unit of Pathology, University of Sheffield, Sheffield, S10 2TN UK; 61 Department of Genetics and Pathology, Pomeranian Medical University, Szczecin, 70-115 Poland; 62 Department of Medical Epidemiology and Biostatistics, Karolinska Institutet, Stockholm, SE-171 77 Sweden; 63 Department of Clinical Genetics, Fox Chase Cancer Center, Philadelphia, 19111 Pennsylvania USA; 64 INSERM U1052, CNRS UMR5286, Université Lyon, Centre de Recherche en Cancérologie de Lyon, Lyon, 69373 France; 65 Department of Pathology and Laboratory Diagnostics the Maria Sklodowska Curie Memorial Cancer Center and Institute of Oncology, Warsaw, 44-101 Poland; 66 Department of Pathology, Leiden University Medical Center, Leiden, 2333 The Netherlands; 67 Department of Human Genetics, Leiden University Medical Center, Leiden, 2333 The Netherlands; 68 Oncogenetics Group, University Hospital Vall d’Hebron, Vall d’Hebron Institute of Oncology (VHIO) and Universitat Autònoma de Barcelona, Barcelona, 08035 Spain; 69 Department of Community and Family Medicine, Section of Biostatistics & Epidemiology, The Geisel School of Medicine at Dartmouth, Lebanon, 03755 New Hampshire USA; 70 Department of Medicine, Abramson Cancer Center, Perelman School of Medicine, University of Pennsylvania, Philadelphia, 19104 Pennsylvania USA; 71 Department of Genetics, University of Pretoria, Pretoria, 0083 South Africa; 72 Genomics Center, Centre Hospitalier Universitaire de Québec Research Center, Laval University, Québec City, G1V 4G2 Québec Canada; 73 Department of Immunology, Genetics and Pathology, Uppsala University, Uppsala, 751 05 Sweden; 74 Department of Clinical Genetics, Lund University Hospital, Lund, 221 00 Sweden; 75 Department of Oncology, Rigshospitalet, Copenhagen University Hospital, Copenhagen, 2100 Denmark; 76 Institute for Medical Informatics, Statistics and Epidemiology, University of Leipzig, Leipzig, 04107 Germany; 77 Genomic Medicine, Manchester Academic Health Sciences Centre, Institute of Human Development, Manchester University, Central Manchester University Hospitals NHS Foundation Trust, Manchester, M13 9PL UK; 78 Department of Medicine, Division of Hematology and Oncology, University of California at Los Angeles, David Geffen School of Medicine, Los Angeles, 90095 California USA; 79 Molecular Diagnostic Unit, Hereditary Cancer Program, IDIBELL (Bellvitge Biomedical Research Institute),Catalan Institute of Oncology, Barcelona, 08908 Spain; 80 Division of Cancer Epidemiology and Genetics, National Cancer Institute, Rockville, 20892 Maryland USA; 81 Institute for Medical Biometrics and Epidemiology, University Medical Center Hamburg-Eppendorf, Hamburg, 20246 Germany; 82 Department of Cancer Epidemiology, Clinical Cancer Registry, University Medical Center Hamburg-Eppendorf, Hamburg, 20246 Germany; 83 Breakthrough Breast Cancer Research Centre, The Institute of Cancer Research, London, SW3 6JB UK; 84 Division of Breast Cancer Research, The Institute of Cancer Research, London, SW7 3RP UK; 85 Department of Breast Surgery, Herlev Hospital, Copenhagen University Hospital, Herlev, 2730 Denmark; 86 Masaryk Memorial Cancer Institute and Medical Faculty MU, Brno, 625 00 Czech Republic; 87 Molecular Diagnostics Laboratory, (INRASTES) Institute of Nuclear and Radiological Sciences and Technology, National Centre for Scientific Research ‘Demokritos’, Aghia Paraskevi Attikis, Athens, 153 10 Greece; 88 Departments of Human Genetics and Oncology, Program in Cancer Genetics, McGill University, Montreal, Montreal, H2W 1S6 Quebec Canada; 89 Biostatistics and Informatics Shared Resource, University of Kansas Medical Center, Kansas City, 66160 Kansas USA; 90 Susanne Levy Gertner Oncogenetics Unit, Sheba Medical Center, Tel-Hashomer, 52621 Israel; 91 Department of Laboratory Medicine, Section of Genetic Oncology, University and University Hospital of Pisa, Pisa, 56126 Italy; 92 Division of Cancer Prevention & Control Research, UCLA Schools of Medicine and Public Health, Jonsson Comprehensive Cancer Center, Los Angeles, 90024 California USA; 93 Cancer Risk and Prevention Clinic, Dana-Farber Cancer Institute, Boston, 02215 Massachusetts USA; 94 Division of Genetics and Epidemiology, The Institute of Cancer Research, London, SW7 3RP UK; 95 Women’s Cancer, UCL EGA Institute for Women’s Health, London, WC1E 6AU UK; 96 Centre for Epidemiology and Biostatistics, Melbourne School of Population and Global Health, The University of Melbourne, Melbourne, 3010 Victoria Australia; 97 Cancer Research UK Clinical Trials Unit, The Beatson West of Scotland Cancer Centre, Glasgow, G12 0YN UK; 98 Department of Pathology and Laboratory Medicine, University of Kansas Medical Center, Kansas City, 66160 Kansas USA; 99 Division of Clinical Epidemiology, Royal Victoria Hospital, McGill University, Montreal, H3A 1A1 Québec Canada; 100 Department of Medicine, McGill University, Montreal, H3A 1A1 Québec Canada; 101 Department of Dermatology, Huntsman Cancer Institute, University of Utah School of Medicine, Salt Lake City, 84132 Utah USA; 102 Department of Health Sciences Research, Mayo Clinic, Rochester, 55902 Minnesota USA; 103 Cancer Prevention and Control, Samuel Oschin Comprehensive Cancer Institute, Cedars-Sinai Medical Center, Los Angeles, 90048 California USA; 104 Department of Biomedical Sciences, Community and Population Health Research Institute, Cedars-Sinai Medical Center, Los Angeles, 90048 California USA; 105 Division of Cancer Epidemiology and Genetics, Clinical Genetics Branch, National Cancer Institute, National Institutes of Health, Rockville, 20892 Maryland USA; 106 Department of Genetics and Pathology, Pomeranian Medical University, Szczecin, 70-204 Poland; 107 Environmental Epidemiology of Cancer, Center for Research in Epidemiology and Population Health, INSERM, Villejuif, 94805 France; 108 University Paris-Sud, Villejuif, 91405 France; 109 Molecular Genetics of Breast Cancer, German Cancer Research Center (DKFZ), Heidelberg, 69120 Germany; 110 Center for Genomic Medicine, Rigshospitalet, Copenhagen University Hospital, Copenhagen, 2100 Denmark; 111 Department of Oncology, Department of Public Health and Primary Care, University of Cambridge, Strangeways Research Laboratory, Cambridge, CB1 8RN UK; 112 Saw Swee Hock School of Public Health, National University of Singapore, Singapore, 119077 Singapore; 113 Breast Cancer Research Unit, Cancer Research Institute, University Malaya Medical Centre, Kuala Lumpur, 50603 Malaysia; 114 Cancer Research Initiatives Foundation, Subang Jaya, Selangor, 47500 Malaysia; 115 Department of Gynecology and Gynecologic Oncology, Kliniken Essen-Mitte, Essen, 45136 Germany; 116 Department of Gynecology and Gynecologic Oncology, Dr Horst Schmidt Kliniken Wiesbaden, Wiesbaden, 65199 Germany; 117 Clinical Cancer Genetics, for the City of Hope Clinical Cancer Genetics Community Research Network, Duarte, 91010 California USA; 118 Department of Pathology, Molecular Unit, Herlev Hospital, University of Copenhagen, Copenhagen, 2730 Denmark; 119 Department of Virus, Lifestyle and Genes, Danish Cancer Society Research Center, Copenhagen, DK-2100 Denmark; 120 Department of Gynecology, Rigshospitalet, University of Copenhagen, Copenhagen, 2100 Denmark; 121 Family Cancer Clinic, Netherlands Cancer Institute, Amsterdam, 1006 The Netherlands; 122 Department of Medical Oncology, Family Cancer Clinic, Erasmus MC Cancer Institute, Rotterdam, 3015 The Netherlands; 123 Center for Medical Genetics, NorthShore University Health System, Evanston, 60201 Illinois USA; 124 N.N. Petrov Institute of Oncology, St Petersburg, 197758 Russia; 125 Lombardi Comprehensive Cancer Center, Georgetown University, Washington, 20057 District of Columbia USA; 126 Division of Epidemiology and Prevention, Aichi Cancer Center Research Institute, Aichi, 464-8681 Japan; 127 State Research Institute Centre for Innovative Medicine, Vilnius, LT-01102 Lithuania; 128 Department of Epidemiology, Cancer Prevention Institute of California, Fremont, 94538 California USA; 129 Department of Preventive Medicine, Seoul National University College of Medicine, Seoul, 08826 Korea; 130 Centre for Epidemiology and Biostatistics, University of Melbourne, Melbourne, 3010 Victoria Australia; 131 Women’s Cancer Program at the Samuel Oschin Comprehensive Cancer Institute, Cedars-Sinai Medical Center, Los Angeles, 90048 California USA; 132 Radboud University Medical Centre, Radboud Institute for Health Sciences, Nijmegen, 6500 The Netherlands; 133 Prosserman Centre for Health Research, Lunenfeld-Tanenbaum Research Institute of Mount Sinai Hospital, Toronto, M5G 1X5 Ontario Canada; 134 Division of Epidemiology, Dalla Lana School of Public Health, University of Toronto, Toronto, M5T 3M7 Ontario Canada; 135 Department of Clinical Pathology, Imaging Center, Kuopio University Hospital, Kuopio, 70210 Finland; 136 Cancer Center, Kuopio University Hospital, Kuopio, 70210 Finland; 137 Institute of Clinical Medicine, Pathology and Forensic Medicine, University of Eastern Finland, Kuopio, 70210 Finland; 138 Department of Clinical Molecular Biology, Oslo University Hospital, University of Oslo, 1478 Oslo, Norway; 139 The Hong Kong Hereditary Breast Cancer Family Registry, Cancer Genetics Center, Hong Kong Sanatorium and Hospital, Hong Kong, China; 140 Department of Surgery, The University of Hong Kong, Hong Kong, China; 141 Vesalius Research Center, VIB, Leuven, 3000 Belgium; 142 Department of Oncology, Laboratory for Translational Genetics, University of Leuven, Leuven, 3000 Belgium; 143 Department of Molecular Medicine and Surgery, Karolinska Institutet, Stockholm, SE-171 77 Sweden; 144 Division of Health Sciences, Warwick Medical School, Warwick University, Coventry, CV4 7AL UK; 145 Department of Gynecologic Oncology, The University of Texas MD Anderson Cancer Center, Houston, 77030 Texas USA; 146 Department of Preventive and Predictive Medicine, Unit of Medical Genetics, Fondazione IRCCS (Istituto Di Ricovero e Cura a Carattere Scientifico) Istituto Nazionale Tumori (INT), Milan, 20133 Italy; 147 University of Hawaii Cancer Center, Honolulu, 96813 Hawaii USA; 148 Department of Oncology - Pathology, Karolinska Institutet, Stockholm, SE-171 77 Sweden; 149 National Center for Tumour Diseases, University of Heidelberg, Heidelberg, 69117 Germany; 150 Department of Gynaecology, Radboud University Medical Centre, Nijmegen, 6500 The Netherlands; 151 Department of Preventive Medicine, Kyushu University Faculty of Medical Sciences, Fukuoka, 812-8582 Japan; 152 Anatomical Pathology, The Alfred Hospital, Melbourne, 3004 Victoria Australia; 153 Institute of Cancer Sciences, University of Glasgow, Wolfson Wohl Cancer Research Centre, Beatson Institute for Cancer Research, Glasgow, G61 1BD UK; 154 Division of Gynaecology and Obstetrics, Technische Universität München, Munich, 81675 Germany; 155 Department of Human Genetics, Radboud University Medical Centre, Nijmegen, 6500 The Netherlands; 156 Immunology and Molecular Oncology Unit, Instituto Oncologico Veneto IOV, IRCCS, Padua, 35128 Italy; 157 Department of Cancer Prevention and Control, Roswell Park Cancer Institute, Buffalo, 14263 New York USA; 158 Institute of Population Health, University of Manchester, Manchester, M13 9PL UK; 159 Laboratory Medicine Program, University Health Network, Toronto, M5G 1L7 Ontario Canada; 160 Department of Laboratory Medicine and Pathobiology, University of Toronto, Toronto, M5G 1L7 Ontario Canada; 161 The University of Texas School of Public Health, Houston, 77030 Texas USA; 162 Department of Population Sciences, Beckman Research Institute of City of Hope, Duarte, 91010 California USA; 163 Department of Medicine and Genetics, University of California, San Francisco, 94143 California USA; 164 Department of Gynecological Oncology, Roswell Park Cancer Institute, Buffalo, 14263 New York USA; 165 Department of Medicine, Memorial Sloan-Kettering Cancer Center, New York, 10065 USA; 166 Department of Molecular Genetics, National Institute of Oncology, Budapest, 1122 Hungary; 167 Center for Clinical Cancer Genetics and Global Health, University of Chicago Medical Center, Chicago, 60637 Illinois USA; 168 The Ohio State University and the James Cancer Center, Columbus, 43210 Ohio USA; 169 Department of Epidemiology and Biostatistics, Memorial Sloan Kettering Cancer Center, New York, 10017 USA; 170 Human Genetics Group, Human Cancer Genetics Program, Spanish National Cancer Centre (CNIO), Madrid, 28019 Spain; 171 Biomedical Network on Rare Diseases (CIBERER), Madrid, 28029 Spain; 172 Department of Surgery, Seoul National University College of Medicine, Seoul, 03080 Korea; 173 Department of Obstetrics and Gynecology, Oregon Health and Science University, Portland, 97239 Oregon USA; 174 Knight Cancer Institute, Oregon Health and Science University, Portland, 97239 Oregon USA; 175 IFOM, The FIRC (Italian Foundation for Cancer Research) Institute of Molecular Oncology, Milan, 16 20139 Italy; 176 Department of Obstetrics and Gynecology, Comprehensive Cancer Center, Medical University of Vienna, Vienna, 1090 Austria; 177 Department of Cancer Epidemiology, Moffitt Cancer Center, Tampa, 33606 Florida USA; 178 Channing Division of Network Medicine, Brigham and Women’s Hospital and Harvard Medical School, Boston, 02115 Massachusetts USA; 179 Department of Epidemiology, Harvard TH Chan School of Public Health, Boston, 02115 Massachusetts USA; 180 Laboratory of Cancer Genetics and Tumour Biology, Northern Finland Laboratory Centre NordLab, Oulu, FI-90014 Finland; 181 Department of Clinical Chemistry and Biocenter Oulu, Laboratory of Cancer Genetics and Tumour Biology, University of Oulu, Oulu, FI-90014 Finland; 182 Department of Preventive and Predictive Medicine, Unit of Molecular Bases of Genetic Risk and Genetic Testing, Fondazione IRCCS (Istituto Di Ricovero e Cura a Carattere Scientifico) Istituto Nazionale dei Tumori (INT), Milan, 20133 Italy; 183 Department of Clinical Genetics, Karolinska University Hospital, Stockholm, 171 76 Sweden; 184 Department of Basic Sciences, Shaukat Khanum Memorial Cancer Hospital and Research Centre (SKMCH & RC), Lahore, 54000 Pakistan; 185 Clalit National Israeli Cancer Control Center and Department of Community Medicine and Epidemiology, Carmel Medical Center and B. Rappaport Faculty of Medicine, Haifa, 34362 Israel; 186 Department of Gynaecology and Obstetrics and Centre for Integrated Oncology (CIO), Centre of Familial Breast and Ovarian Cancer, Center for Molecular Medicine Cologne (CMMC), University Hospital of Cologne, Cologne, 50931 Germany; 187 Department of Chronic Disease Epidemiology, Yale School of Public Health, New Haven, 06510 Connecticut USA; 188 Division of Gynecologic Oncology, NorthShore University HealthSystem, Evanston, 60201 Illinois USA; 189 Division of Public Health Sciences, Program in Epidemiology, Fred Hutchinson Cancer Research Center, Seattle, 98109 Washington USA; 190 Department of Epidemiology, University of Washington, Seattle, 98109 Washington USA; 191 National Cancer Institute, Bangkok, 10400 Thailand; 192 Research Oncology, Guy’s Hospital, King’s College London, London, SE1 9RT UK; 193 Department of Community and Family Medicine, Duke University Medical Center, Durham, 27710 North Carolina USA; 194 Cancer Control and Population Sciences, Duke Cancer Institute, Durham, 27710 North Carolina USA; 195 Netherlands Cancer Institute, Antoni van Leeuwenhoek Hospital, Amsterdam, 1066 CX The Netherlands; 196 Division of Molecular Gyneco-Oncology, Department of Gynaecology and Obstetrics, University Hospital of Cologne, Cologne, 50676 Germany; 197 Center for Integrated Oncology, University Hospital of Cologne, Cologne, 50676 Germany; 198 Center for Molecular Medicine, University Hospital of Cologne, Cologne, 50676 Germany; 199 Center of Familial Breast and Ovarian Cancer, University Hospital of Cologne, Cologne, 50676 Germany; 200 Taiwan Biobank, Institute of Biomedical Sciences, Academia Sinica, Taipei, 115 Taiwan; 201 School of Public Health, China Medical University, Taichung, 404 Taiwan; 202 Department of Health Research and Policy - Epidemiology, Stanford University School of Medicine, Stanford, 94305 California USA; 203 Unité Mixte de Génétique Constitutionnelle des Cancers Fréquents, Hospices Civils de Lyon – Centre Léon Bérard, Lyon, 69008 France; 204 INSERM U1052, CNRS UMR5286, Université Lyon 1, Centre de Recherche en Cancérologie de Lyon, Lyon, 69003 France; 205 Department of Pathology, University of Melbourne, Parkville, 3010 Victoria Australia; 206 Division of Clinical Genetics, Department of Clinical and Experimental Medicine, Linköping University, Linköping, 581 83 Sweden; 207 Institut Curie, Department of Tumour Biology, Paris, France; Department of Tumour Biology, Institut Curie, Paris, France; 208 Institut Curie, INSERM U830, Paris, 75248 France; 209 Université Paris Descartes, Sorbonne Paris Cité, Paris, 75270 France; 210 Department of Human Genetics, Institute of Human Genetics, University Hospital Heidelberg, Heidelberg, 69120 Germany; 211 Department of Genetics, Portuguese Oncology Institute, Porto, 4200-072 Portugal; 212 Biomedical Sciences Institute (ICBAS), Porto University, Porto, 4099-002 Portugal; 213 Department of Epidemiology, Mailman School of Public Health, Columbia University, New York, 10027 USA; 214 Department of Clinical Genetics, Odense University Hospital, Odense C, 5000 Denmark; 215 UO Anatomia Patologica, Ospedale di Circolo-Università dell’Insubria, Varese, 21100 Italy; 216 Latvian Biomedical Research and Study Centre, Riga, LV-1067 Latvia Sweden; 217 Immunology and Molecular Oncology Unit, Istituto Oncologico Veneto IOV - IRCCS (Istituto Di Ricovero e Cura a Carattere Scientifico), Padua, 64 - 35128 Italy; 218 Department of Molecular Virology, Immunology and Medical Genetics, The Ohio State University, Columbus, 43210 Ohio USA; 219 Wellcome Trust Centre for Human Genetics and Oxford Biomedical Research Centre, University of Oxford, Oxford, OX3 7BN UK; 220 Institute of Human Genetics, Pontificia Universidad Javeriana, Cra. 7 #40-62, Bogota, Colombia; 221 Department of Medical Oncology, Beth Israel Deaconess Medical Center, Boston, 02215 Massachusetts USA; 222 Department of Clinical Genetics, Erasmus University Medical Center, Rotterdam, 3015 CE The Netherlands; 223 Department of Gynecology, Family Cancer Clinic, Erasmus MC Cancer Institute, Rotterdam, 3015 CE The Netherlands; 224 Division of Gynecological Oncology, Department of Oncology, University Hospitals Leuven, Leuven, B-3000 Belgium; 225 University Hospital Ulm, Ulm, 89069 Germany; 226 Division of Cancer Epidemiology and Genetics, National Cancer Institute, Bethesda, 20892 Maryland USA; 227 Department of General Medical Oncology, Multidisciplinary Breast Center, University Hospitals Leuven, Leuven, B-3000 Belgium; 228 Molecular Diagnostics Laboratory, IRRP, National Centre for Scientific Research ‘Demokritos’, Athens, 153 10 Greece; 229 Cancer Research Initiatives Foundation, Sime Darby Medical Centre, Subang Jaya, 47500 Malaysia; 230 University Malaya Cancer Research Institute, Faculty of Medicine, University Malaya Medical Centre, University Malaya, Kuala Lumpur, 59100 Malaysia; 231 Department of Surgery, Tri-Service General Hospital, National Defense Medical Center, Taipei, 114 Taiwan; 232 Shanghai Center for Disease Control and Prevention, Shanghai, China; 233 Division of Population Sciences, Cancer Epidemiology Program, H. Lee Moffitt Cancer Center & Research Institute, Tampa, 33612 Florida USA; 234 Department of Laboratory Medicine and Pathology, Mayo Clinic, Rochester, 55905 Minnesota USA; 235 Present Address: Women’s Cancer Program at the Samuel Oschin Comprehensive Cancer Institute, Cedars-Sinai Medical Center, Los Angeles, CA USA; 236 Present Address: The Center for Bioinformatics and Functional Genomics, Cedars-Sinai Medical Center, Los Angeles, CA USA; 237 Service de Génétique, CHU de Besançon, Besançon, 25030 France; 238 Oncogénétique, Institut Bergonié, 229 cours de l’Argonne, Bordeaux, 33076 France; 239 Centre François Baclesse, 3 avenue Général Harris, Caen, 14000 France; 240 Laboratoire de Génétique Chromosomique, Hôtel Dieu Centre Hospitalier, Chambéry, BP 1125 France; 241 Centre Jean Perrin, Clermont-Ferrand cedex, BP 392 France; 242 Centre de Lutte Contre le Cancer Georges François Leclerc, 1 rue Professeur Marion, Dijon Cedex, BP 77 980 France; 243 Département de Génétique, CHU de Grenoble, Grenoble Cedex 9, BP 217 France; 244 Centre Oscar Lambret, 3 rue Frédéric Combemale, 59020, Lille cedex, BP307 France; 245 Department of Medical Oncology, CHU Dupuytren, Limoges, 87042 France; 246 Service de Génétique Moléculaire et Clinique, Hôpital Edouard Herriot, 5 place d’Arsonval, Lyon cedex 03, 69437 France; 247 Centre Léon Bérard, 28 rue Laënnec, Lyon, 69437 France; 248 Unité de Prévention et d’Epidémiologie Génétique, Centre Léon Bérard, 28 rue Laënnec, Lyon, 69437 France; 249 Biopathologie, Centre Léon Bérard, 28 rue Laënnec, Lyon, 69437 France; 250 Département Oncologie Génétique, Prévention et Dépistage, Institut Paoli-Calmettes, 232 boulevard Sainte-Marguerite, Marseille, 13009 France; 251 Unité d’Oncogénétique, CHU Arnaud de Villeneuve, Montpellier Cedex 5, 34295 France; 252 Laboratoire de génétique médicale, Nancy Université, Centre Hospitalier Régional et Universitaire, Rue du Morvan, cedex 1, 54511 Vandoeuvre-les-Nancy France; 253 Service d’Oncogénétique, Centre René Gauducheau, Boulevard Jacques Monod, Nantes Saint Herblain Cedex, 44805 France; 254 Centre Antoine Lacassagne, 33 Avenue de Valombrose, Nice, 06100 France; 255 Service de Génétique, Institut Curie, 26, rue d’Ulm, Paris Cedex 05, 75248 France; 256 Inserm U830, Université INSERM U830, centre de recherche de l’Institut Curie, Paris, 75013 France; 257 Inserm U900, Institut Curie, Mines ParisTech, PSL University, 26 rue d’Ulm, Paris Cedex 05, 75248 France; 258 Département de Génétique, Groupe Hospitalier Pitié-Salpétrière, 47-83 boulevard de l’Hôpital, Paris, 75013 France; 259 Service de Génétique Clinique Chromosomique et Moléculaire, Hôpital Nord, CHU Saint Etienne, St Etienne, 42055 Cedex 2 France; 260 Unité d’Oncogénétique, Centre Paul Strauss, 3 rue de la Porte de l’Hôpital, Strasbourg, BP30042 France; 261 Oncogénétique, Institut Claudius Regaud, 1 avenue Irène Joliot-Curie, Toulouse cedex 9, 31059 France; 262 Hôpital Bretonneau - CHU de Tours, 2 boulevard Tonnelé, Tours cedex, 37004 France; 263 Service de Génétique, Institut Gustave Roussy, 39, rue Camille Desmoulins, Villejuif Cedex, 94805 France; 264 North of Scotland Regional Genetics Service, NHS Grampian & University of Aberdeen, Foresterhill, AB24 3AA Aberdeen UK; 265 and Department of Medical Genetics, Northern Ireland Regional Genetics Centre, Belfast Health and Social Care Trust, Queens University Belfast, Belfast, BT9 7BL UK; 266 Clinical Genetics Department, St Michael’s Hospital, Bristol, BS2 8EG UK; 267 All Wales Medical Genetics Services, University Hospital of Wales, Cardiff, CF14 4XW UK; 268 Academic Unit of Clinical and Molecular Oncology, Trinity College Dublin, Dublin, 2 Ireland; 269 St James’s Hospital, Dublin, 8 Ireland; 270 South East of Scotland Regional Genetics Service, Western General Hospital, Edinburgh, EH4 2XU UK; 271 North West Thames Regional Genetics Service, Kennedy-Galton Centre, Harrow, HA1 3UJ UK; 272 Leicestershire Clinical Genetics Service, University Hospitals of Leicester NHS Trust, Leicester, LE1 5WW UK; 273 Department of Clinical Genetics, Alder Hey Hospital, Eaton Road, Liverpool, L12 2AP UK; 274 Genetic Medicine, Manchester Academic Health Sciences Centre, Central Manchester University Hospitals NHS Foundation Trust, Manchester, M13 9WL UK; 275 North East Thames Regional Genetics Service, Great Ormond Street Hospital for Children NHS Trust, London, WC1N 3JH UK; 276 Nottingham Clinical Genetics Service, Nottingham University Hospitals NHS Trust, Nottingham, NG5 1PB UK; 277 Institute of Genetic Medicine, Centre for Life, Newcastle Upon Tyne Hospitals NHS Trust, Newcastle upon Tyne, NE7 7DN UK; 278 Oxford Regional Genetics Service, Churchill Hospital, Oxford, OX3 7LE UK; 279 Sheffield Clinical Genetics Service, Sheffield Children’s Hospital, Sheffield, S10 2TH UK; 280 South West Thames Regional Genetics Service, St.Georges Hospital, Cranmer Terrace, Tooting, London, SW17 0RE UK; 281 All Wales Medical Genetics Services, Singleton Hospital, Swansea, SA2 8QA UK; 282 All Wales Medical Genetics Service, Glan Clwyd Hospital, Rhyl, LL18 5UJ UK; 283 Department of Epidemiology, Netherlands Cancer Institute, PO Box 90203, Amsterdam, 1006 BE The Netherlands; 284 Family Cancer Clinic, Netherlands Cancer Institute, PO Box 90203, Amsterdam, 1006 BE The Netherlands; 285 Division of Psychosocial Research and Epidemiology, Netherlands Cancer Institute, PO Box 90203, Amsterdam, 1006 BE The Netherlands; 286 Department of Radiotherapy, Netherlands Cancer Institute, PO Box 90203, Amsterdam, 1006 BE The Netherlands; 287 Department of Clinical Genetics, Family Cancer Clinic, Erasmus University Medical Center, PO Box 2040, Amsterdam, 3000 CA The Netherlands; 288 Department of Medical Oncology, Family Cancer Clinic, Erasmus MC Cancer Institute, PO Box 5201, Rotterdam, 3008 AE The Netherlands; 289 Department of Pathology, Family Cancer Clinic, Erasmus University Medical Center, PO Box 2040, Amsterdam, 3000 CA The Netherlands; 290 Department of Radiology, Family Cancer Clinic, Erasmus University Medical Center, PO Box 2040, Amsterdam, 3000 CA The Netherlands; 291 Department of Clinical Genetics, Leiden University Medical Center, PO Box 9600, Leiden, 2300 RC The Netherlands; 292 Department of Surgery, Leiden University Medical Center, PO Box 9600, Leiden, 2300 RC The Netherlands; 293 Department of Human Genetics, Radboud University Medical Center, PO Box 9101, Nijmegen, 6500 HB The Netherlands; 294 Department of Medical Genetics, University Medical Center Utrecht, Utrecht, PO Box 85090, 3508 AB The Netherlands; 295 Department of Oncological and Endocrine Surgery, University Medical Center Utrecht, PO Box 85090, Utrecht, 3508 AB The Netherlands; 296 Department of Clinical Genetics, Academic Medical Center, PO Box 22700, Amsterdam, 1100 DE The Netherlands; 297 Department of Clinical Genetics, VU University Medical Center, PO Box 7057, Amsterdam, 1007 MB The Netherlands; 298 Department of Clinical Genetics and GROW, School for Oncology and Developmental Biology, Maastricht University Medical Center, PO Box 5800, Maastricht, 6202 AZ The Netherlands; 299 Department of Genetics, University Medical Center Groningen, PO Box 30.001, Groningen, 9700 RB The Netherlands; 300 Department of Gynaecology, University Medical Center Groningen, PO Box 30.001, Groningen, 9700 RB The Netherlands; 301 Department of Epidemiology, University Medical Center Groningen, PO Box 30.001, Groningen, 9700 RB The Netherlands; 302 The Netherlands Foundation for Detection of Hereditary Tumours, University Medical Center, Poortgebouw Zuid, Leiden, 2333 AA The Netherlands; 303 The Netherlands Comprehensive Cancer Organization (IKNL), Location Amsterdam, IJsbaanpad 9-11, Amsterdam, 1076 CV The Netherlands; 304 The nationwide network and registry of histo- and cytopathology in the Netherlands (PALGA), Randhoeve 225A, Houten, 3995 GA The Netherlands; 305 Pathology Department, Peter MacCallum Cancer Centre, Melbourne, 3002 Victoria Australia; 306 Department of Medicine, Familial Cancer Service, Westmead Hospital, Westmead, 2145 New South Wales Australia; 307 Breast Cancer Laboratory, Walter and Eliza Hall Institute, PO Royal Melbourne Hospital, Parkville, 3050 Victoria Australia; 308 Medical Psychology, University of Sydney, Sydney, 2006 New South Wales Australia; 309 Peter MacCallum Cancer Centre, East Melbourne, 3002 Victoria Australia; 310 Sir Peter MacCallum Cancer Centre Department of Oncology, University of Melbourne, Parkville, 3052 Victoria Australia; 311 Department of Surgery and Cancer, Ovarian Cancer Action Research Centre, Imperial College London, London, W12 0HS UK; 312 Department of Biochemistry and Molecular Biology, University of Melbourne, Parkville, 3052 Victoria Australia; 313 Department of Gynaecological Oncology, Westmead Institute for Cancer Research, Westmead Hospital, Westmead, 2145 New South Wales Australia

**Keywords:** Cancer genetics, Genetic association study, Epigenetics, Breast cancer

Correction to: *Nature Communications* 10.1038/ncomms12675, published online 7 September 2016

In this article the author name Rikki Cannioto was incorrectly written as Rikki Canniotto. The original article has been corrected.

