# Supplementary material for: Functional mechanisms underlying pleiotropic risk alleles at the 19p13.1 breast–ovarian cancer susceptibility locus
Source: Nat Commun. 2016 Sep 7;7:12675. doi: 10.1038/ncomms12675 (PMC5023955; doi:10.1038/ncomms12675)
Supplement: Supplementary Information — Supplementary Figures 1-4, Supplementary Tables 1-10 and Supplementary References. (PDF 1359 kb) [file 41467_2016_BFncomms12675_MOESM1635_ESM.pdf]

SUPPLEMENTARY INFORMATION

Supplementary Figure 1.

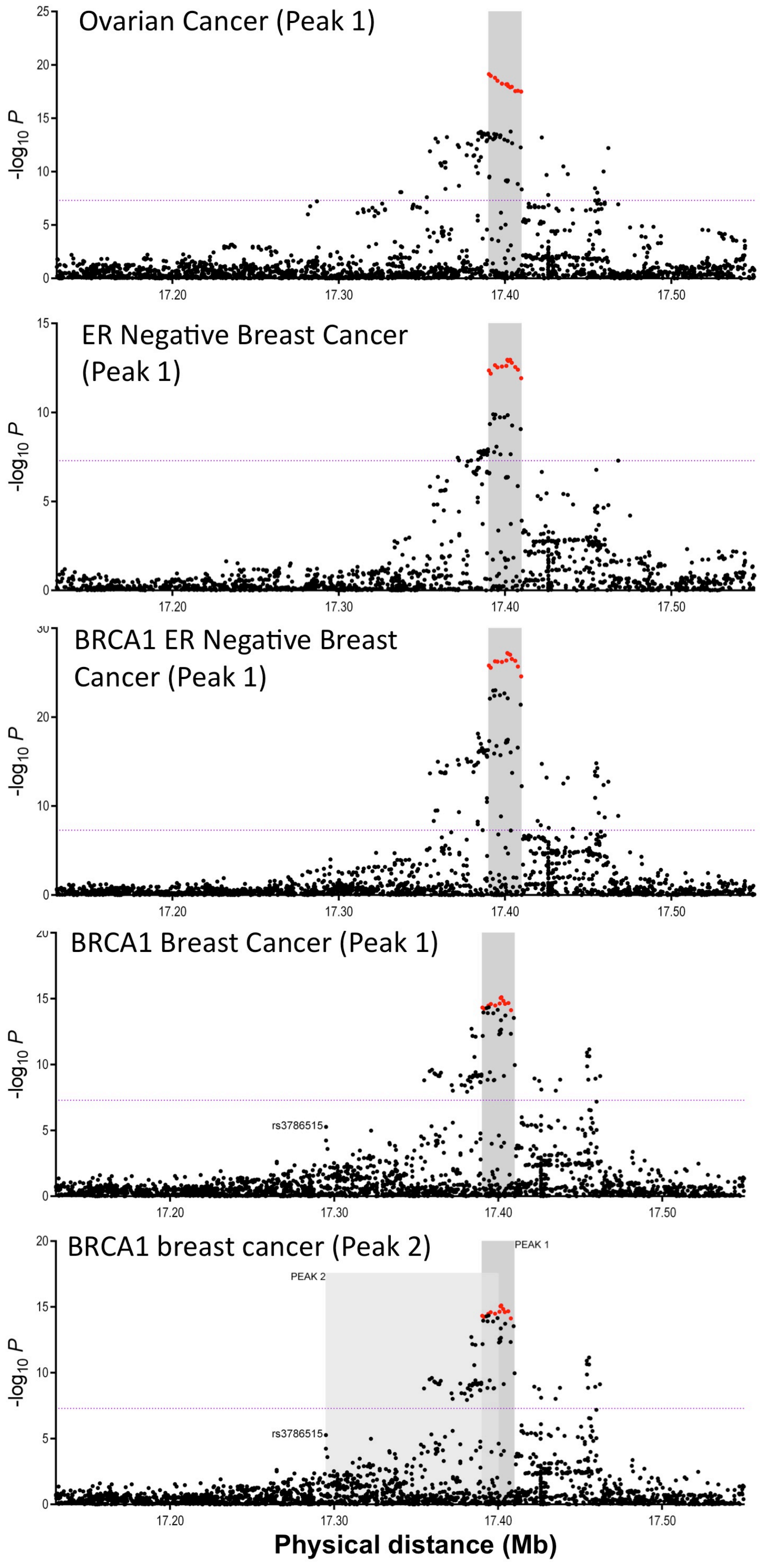

Phenotype-specific regional association plots. Candidate causal alleles are highlighted in red.

Supplementary Figure 2.

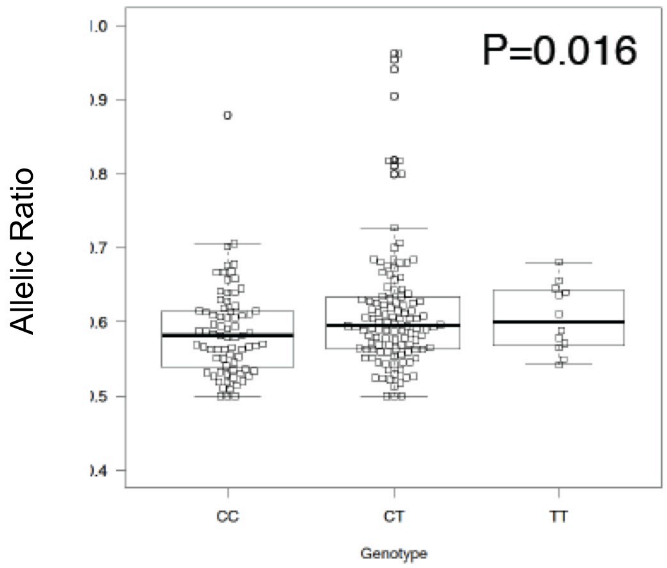

Allele specific expression (ASE) analysis in breast cancer tissues. Allele specific expression analysis in 380 breast cancers identified a significant association between rs4808616 and *ABHD8* allelic ratio (F-test P=0.016).

Supplementary Figure 3.

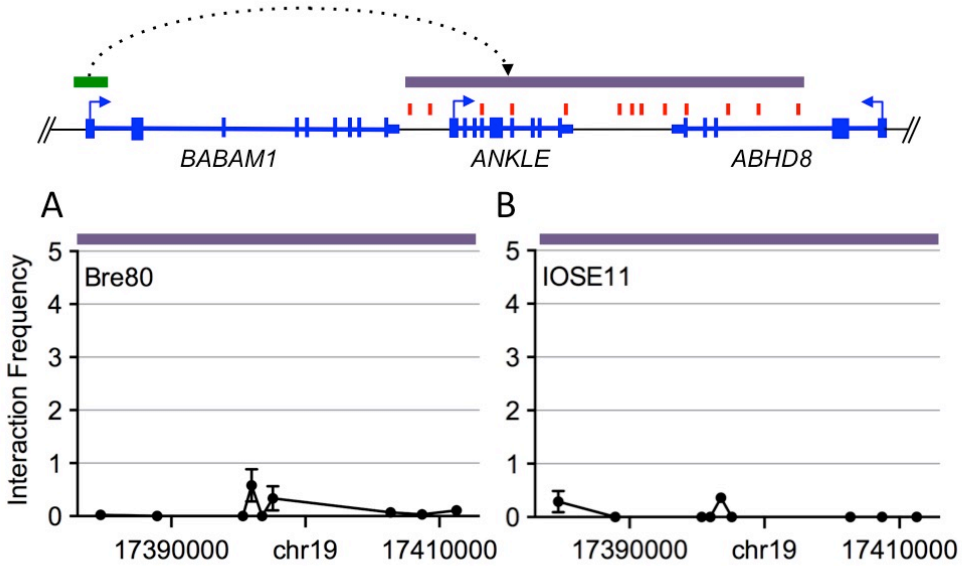

Supplementary Figure 3. 3C analysis of interactions between the *BABAM1* promoter and candidate causal SNPs. 3C performed in (a) normal breast cells and (b) normal ovarian epithelial cells did not detect any significant interactions between a fragment containing all risk SNPs, and the promoter of *BABAM1*.

Supplementary Figure 4.

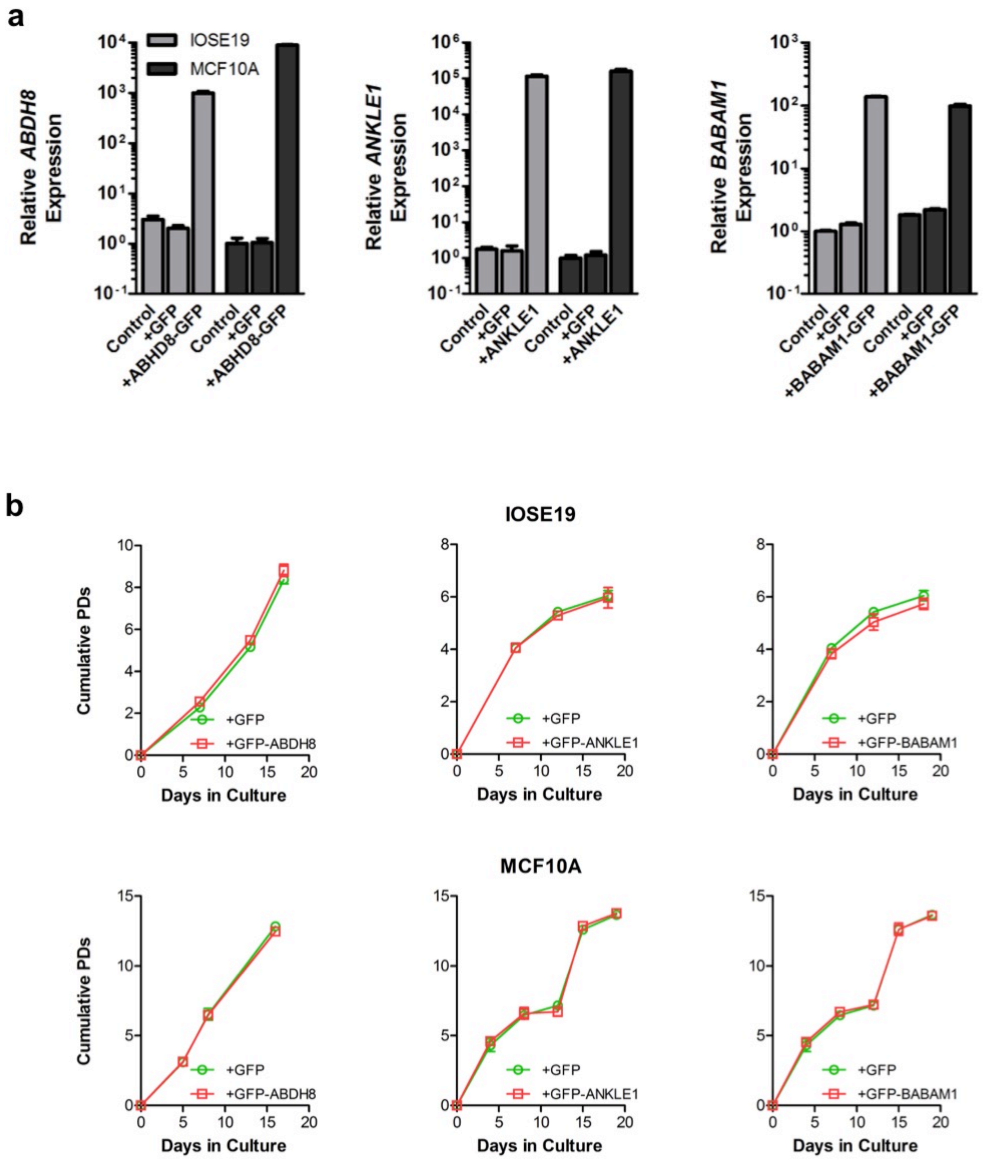

Supplementary Figure 4. Analysis of *ABHD8, ANKLE1* and *BABAM1* overexpression models. (a) Gene-specific qPCR analysis of *ABHD8, ANKLE1* and *BABAM1* overexpression in MCF10A and IOSE19 cells. (b) Growth curve analysis, expression of these genes did not impact cellular proliferation.

Supplementary Table 1. Number of samples using in the analysis. ER, estrogen receptor; TN, triple negative; OC, ovarian cancer. *Used in competing risk analyses

**Study/ethnic group**						
**BCAC**	**Controls**	**Cases**	**ER-negative**	**ER-positive**	**TN**	**ER-/HER2+**
European	42,599	46,451	7,435	27,074	2,837	932
Asian	6,624	6,269	1,623	3,374	NA	NA
African-American	932	1117	NA	NA	NA	NA
**BRCA1 (CIMBA)**	**Unaffected**	**Breast Cancer**	**ER-negative**	**Ovarian Cancer***		
European	7,797	7,455	2,639	1,724		
**OCAC**	**Controls**	**Cases**	**High grade serous OC**	**Endometrioid OC**	**Clear Cell OC**	**Mucinous OC**
European	30,845	15,438	9,627	2,152	1,016	1,003

Supplementary Table 2. Associations for ovarian cancer, by histological subtype. Position from NCBI build 37, χ^2-test p-values shown.

		Endometrioid			Clear Cell			Mucinous		
**SNP**	**Position (Chr19)**	**OR**	**95% CI**	**P-Value**	**OR**	**95% CI**	**P-Value**	**OR**	**95% CI**	**P-Value**
rs4808075	17390291	0.96	0.90-1.03	0.29	1.05	0.95-1.16	0.34	0.93	0.85-1.01	0.08
rs10419397	17391328	0.96	0.90-1.03	0.30	1.05	0.95-1.16	0.33	0.93	0.85-1.01	0.08
rs56069439	17393925	0.97	0.90-1.04	0.33	1.05	0.95-1.16	0.32	0.92	0.85-1.01	0.08
rs4808076	17395401	0.97	0.90-1.04	0.32	1.05	0.95-1.16	0.32	0.92	0.85-1.01	0.07
rs111961716	17398085	0.97	0.90-1.04	0.36	1.05	0.96-1.16	0.29	0.92	0.85-1.01	0.07
rs113299211	17400765	0.97	0.90-1.04	0.35	1.05	0.95-1.16	0.30	0.92	0.85-1.01	0.07
rs67397200	17401404	0.97	0.90-1.04	0.34	1.05	0.95-1.16	0.32	0.92	0.84-1.00	0.06
rs61494113	17401859	0.97	0.90-1.04	0.33	1.05	0.95-1.16	0.34	0.92	0.84-1.00	0.05
rs4808616	17403033	0.97	0.90-1.04	0.34	1.05	0.95-1.16	0.31	0.92	0.85-1.00	0.07
rs55924783	17404072	0.97	0.90-1.04	0.32	1.05	0.95-1.16	0.31	0.92	0.84-1.00	0.06
rs28473003	17406167	0.96	0.90-1.03	0.31	1.05	0.95-1.16	0.33	0.92	0.84-1.00	0.06
rs13343778	17407695	0.97	0.90-1.04	0.34	1.05	0.95-1.16	0.34	0.92	0.84-1.00	0.06
rs10424198	17409671	0.98	0.91-1.05	0.52	1.05	0.95-1.15	0.38	0.91	0.83-0.99	0.04

Supplementary Table 3. Pair-wise correlation between previously identified candidate causal variants for breast and ovarian cancer and BRCA1 mutation carriers with breast cancer (Bolton et al, 2010, *Nat Genet* **42**, 880-4; Antoniou et al 2010, *Nat Genet* **42**, 885-92; Stevens, K.N. *et al. Cancer Res* 2012 **72**, 1795-803) and new genetic risk variants identified in the current study. The table shows pairwise r^2 correlations between previous GWAS hits and current top SNPs. Data are from 1000 Genomes, Phase 3, version 5.

	rs3786515 [1]	rs8170 [3a]	rs8100241 [4a]	rs2363956 [4]	rs67397200[2]
rs3786515 [1]		0.00	0.00	0.00	0.00
rs8170 [3]	0.00		0.26	0.26	0.55
rs8100241 [4a]	0.00	0.26		1.00	0.47
rs2363956 [4]	0.00	0.26	1.00		0.47
rs67397200 [2]	0.00	0.55	0.47	0.47	

[1] Strongest breast cancer risk associated SNP in PEAK 2 identified in the current analysis from the analysis of BRCA1 mutation carriers; [2] Strongest associated SNP in PEAK 1 identified in the current analysis from the meta-analysis of breast cancer risk for BRCA1 mutation carriers and ER-negative breast cancer from BCAC; [3] Strongest risk association with triple negative breast cancer risk identified by Stevens, K.N. *et al.* 2012 [1], and with breast cancer risk for *BRCA1* mutation carriers identified by Antoniou et al 2010 [2] [4] Strongest risk associated SNP for high grade serous ovarian cancer identified previously by Bolton et al, 2010 [3] and Antoniou et al 2010 [2] for breast cancer risk for *BRCA1* mutation carriers (rs8100241 and rs2363956 are perfectly correlated) [a]The most parsimonious model for the association with BRCA1 breast cancer risk in Antoniou et al 2010 [2], included SNPs rs8170 and rs8100241 (or rs2363656).

Supplementary Table 4. Associations at P<10^{-4} with ER-negative breast cancer (BCAC), *BRCA1* breast cancer risk and serous OC. OR, odds ratio. # Results only for genotyped SNPs; † Results only for genotyped SNPs, competing risks models. ER, estrogen receptor; TN, triple negative; BC, breast cancer; OC, ovarian cancer.

	ER-positive BC			Triple Negative BC (N cases=2837)			ER-negative and HER2-positive BC (N cases=932)			TN vs ER-/HER2+ BC case only	ER-negative *BRCA1*-associated BC #			*BRCA1* associated OC †		
**SNP**	**OR**	**95%CI**	**p-trend**	**OR**	**95%CI**	**p-trend**	**OR**	**95%CI**	**p-trend**	**p-diff**	**OR**	**95%CI**	**p-trend**	**OR**	**95%CI**	**p-trend**
**PEAK 1**																
rs4808075	1.00	0.97-1.02	0.90	1.29	1.21-1.38	6.4E-14	1.00	0.90-1.11	0.97	2.94E-05						
rs10419397	1.00	0.97-1.02	0.89	1.29	1.21-1.38	8.07E-14	1.00	0.90-1.11	0.99	2.96E-05						
rs56069439	1.00	0.97-1.02	0.89	1.30	1.21-1.39	2.97E-14	1.01	0.91-1.12	0.89	3.43E-05						
rs4808076	1.00	0.97-1.02	0.89	1.29	1.21-1.38	4.27E-14	1.01	0.91-1.12	0.91	3.53E-05						
rs111961716	1.00	0.97-1.02	0.84	1.29	1.21-1.38	4.15E-14	1.01	0.91-1.11	0.92	3.37E-05						
rs113299211	1.00	0.97-1.02	0.80	1.30	1.21-1.39	3.07E-14	1.00	0.91-1.11	0.93	3.07E-05						
rs67397200	1.00	0.97-1.02	0.80	1.30	1.22-1.39	1.29E-14	1.01	0.91-1.11	0.91	2.11E-05	1.22	1.17-1.28	3.28E-17	1.11	1.02-1.21	0.002
rs61494113	1.00	0.97-1.02	0.83	1.30	1.21-1.39	1.62E-14	1.01	0.91-1.12	0.88	2.16E-05	1.25	1.19-1.32	3.00E-17	1.11	1.02-1.21	0.017
rs4808616	1.00	0.97-1.02	0.76	1.30	1.21-1.39	1.24E-14	1.01	0.91-1.12	0.89	2.36E-05	1.25	1.18-1.31	1.22E-16	1.11	1.02-1.21	0.026
rs55924783	1.00	0.97-1.02	0.76	1.30	1.21-1.39	1.70E-14	1.00	0.91-1.11	0.94	2.27E-05						
rs28473003	1.00	0.97-1.02	0.76	1.30	1.21-1.39	2.20E-14	1.00	0.90-1.11	0.99	1.60E-05						
rs13343778	1.00	0.97-1.02	0.83	1.30	1.21-1.39	2.62E-14	0.99	0.90-1.10	0.91	1.09E-05						
rs10424198	0.99	0.97-1.02	0.53	1.30	1.22-1.39	2.00E-14	0.99	0.89-1.10	0.88	6.61E-06						
**PEAK 2**																
rs3786514	1.01	0.99-1.03	0.51	0.99	0.93-1.06	8.54E-01	1.03	0.94-1.13	0.56	5.79E-01	1.13	1.07-1.18	1.48E-06	1.02	0.94-1.10	0.670
rs3786515	1.01	0.99-1.04	0.24	0.99	0.93-1.06	8.80E-01	1.01	0.92-1.11	0.86	9.26E-01	1.14	1.09-1.20	7.43E-08	0.99	0.92-1.07	0.860
rs891205	1.00	0.97-1.02	0.71	1.12	1.04-1.19	1.47E-03	0.99	0.90-1.09	0.80	3.26E-02	1.12	1.07-1.18	4.86E-06	1.08	1.00-1.17	0.060
rs7247493	1.00	0.97-1.02	0.69	1.11	1.04-1.19	1.86E-03	0.99	0.90-1.09	0.82	3.70E-02	1.12	1.07-1.18	6.20E-06	1.08	1.00-1.17	0.051
rs7246243	0.99	0.97-1.02	0.50	1.12	1.05-1.19	8.56E-04	0.99	0.90-1.09	0.85	4.96E-02						
rs4464206	0.99	0.97-1.02	0.64	1.12	1.05-1.20	1.15E-03	1.00	0.90-1.10	0.93	4.05E-02	1.14	1.08-1.20	4.36E-07	1.08	1.00-1.17	0.063
c19_pos17261271	0.99	0.97-1.01	0.50	0.89	0.83-0.95	5.48E-04	1.02	0.93-1.12	0.66	1.48E-02	0.89	0.84-0.93	2.17E-06	1.06	0.98-1.14	0.157

Supplementary Table 5. Significant (P<0.05, linear regression) eQTL results for TCGA HGSOC. Grey fields are significant at P<0.01

SNP	Gene	P-value	FDR
Peak 1 SNPs (genotyped)			
rs4808616	ABHD8	3.0E-05	0.14
rs4808616	NXNL1	0.004	0.42
rs4808616	PGLS	0.010	0.49
rs4808616	PLVAP	0.018	0.51
rs4808616	FAM129C	0.031	0.56

Supplementary Table 6. Pathway analysis of *BABAM1* overexpression models. Analyses were performed using Ingenuity Pathway Analysis, p-values from right-tailed Fisher's Exact Tests.

Analysis	Cell Line	Pathway	P-Value
Canonical Pathways	IOSE19	EIF2 Signaling	6.46E-08
		Regulation of eIF4 and p70S6K Signaling	2.75E-05
		mTOR Signaling	7.41E-05
		Superoxide Radicals Degradation	7.24E-03
		Sucrose Degradation V (Mammalian)	9.55E-03
		Prostanoid Biosynthesis	1.10E-02
		Gluconeogenesis I	2.75E-02
		Glycolysis I	2.88E-02
		Systemic Lupus Erythematosus Signaling	2.95E-02
		Pyrimidine Ribonucleotides Interconversion	3.31E-02
	MCF10A	EIF2 Signaling	3.09E-07
		Regulation of eIF4 and p70S6K Signaling	7.41E-05
		mTOR Signaling	2.00E-04
		Asparagine Biosynthesis I	1.55E-03
		eNOS Signaling	1.74E-02
		NRF2-mediated Oxidative Stress Response	3.09E-02
		ILK Signaling	3.31E-02
		Estrogen-mediated S-phase Entry	3.63E-02
		Antiproliferative Role of TOB in T Cell Signaling	3.89E-02
		Role of p14/p19ARF in Tumor Suppression	4.57E-02
Disease and Functions	IOSE19	Connective Tissue Disorders	1.53E-06-3.92E-02
		Inflammatory Disease	1.53E-06-4.62E-02
		Skeletal and Muscular Disorders	1.53E-06-3.92E-02
		Energy Production	4.19E-06-4.96E-02
		Gastrointestinal Disease	4.19E-06-2.75E-02
		Hepatic System Disease	4.19E-06-1.44E-02
		Immunological Disease	4.92E-06-4.04E-02
		Antimicrobial Response	2.03E-05-6.04E-03
		Inflammatory Response	2.03E-05-4.27E-02
		Free Radical Scavenging	2.36E-04-2.28E-02
	MCF10A	Dermatological Diseases and Conditions	3.62E-08-4.67E-02
		Developmental Disorder	3.62E-08-2.74E-02
		Organismal Injury and Abnormalities	3.62E-08-4.67E-02
		Embryonic Development	2.81E-07-4.96E-02

		Hair and Skin Development and Function	2.81E-07-4.96E-02
		Organ Development	2.81E-07-4.96E-02
		Organismal Development	2.81E-07-4.96E-02
		Tissue Development	2.81E-07-4.96E-02
		Immunological Disease	4.25E-07-4.37E-02
		Inflammatory Disease	4.25E-07-1.07E-02

Supplementary Table 7. Pathway analysis of *ANKLE1* overexpression models. Analyses were performed using Ingenuity Pathway Analysis, p-values from right-tailed Fisher's Exact Tests.

Analysis	Cell Line	Pathway	P-Value
Canonical Pathways	IOSE19	EIF2 Signaling	5.01E-25
		Regulation of eIF4 and p70S6K Signaling	1.02E-08
		mTOR Signaling	8.51E-08
		Hepatic Fibrosis / Hepatic Stellate Cell Activation	1.78E-07
		ILK Signaling	1.35E-04
		Guanine and Guanosine Salvage I	4.17E-04
		Cell Cycle: G2/M DNA Damage Checkpoint Regulation	2.00E-03
		Aryl Hydrocarbon Receptor Signaling	2.40E-03
		Arsenate Detoxification I (Glutaredoxin)	2.45E-03
		Vitamin-C Transport	2.63E-03
	MCF10A	EIF2 Signaling	2.09E-10
		Glutathione Redox Reactions I	1.23E-03
		Pyrimidine Deoxyribonucleotides De Novo Biosynthesis I	2.45E-03
		Cell Cycle Control of Chromosomal Replication	3.31E-03
		Role of p14/p19ARF in Tumor Suppression	3.80E-03
		Spliceosomal Cycle	6.17E-03
		Putrescine Biosynthesis III	6.17E-03
		NADH Repair	9.12E-03
		Glutathione Redox Reactions II	9.12E-03
		Regulation of eIF4 and p70S6K Signaling	1.07E-02
Disease and Functions	IOSE19	Cellular Growth and Proliferation	2.43E-27-8.29E-04
		Cell Death and Survival	9.05E-21-6.92E-04
		Cancer	3.79E-17-8.58E-04
		Organismal Survival	1.2E-16-8.18E-04
		Cell Cycle	2.36E-16-6.56E-04
		Cellular Movement	5.02E-15-8.42E-04
		Cardiovascular Disease	1.83E-14-8.06E-04
		Cellular Development	3.65E-14-8.29E-04
		Cardiovascular System Development and Function	3.37E-13-7.91E-04
		Organismal Development	3.37E-13-8.38E-04
	MCF10A	RNA Post-Transcriptional Modification	3.18E-07-2.83E-02
		Cancer	3.36E-06-3.04E-02
		Organismal Injury and Abnormalities	3.36E-06-3.01E-02
		Reproductive System Disease	3.36E-06-2.01E-02
		Nucleic Acid Metabolism	9.32E-06-2.53E-02
		Small Molecule Biochemistry	9.32E-06-3.04E-02

		Cell Death and Survival	1.56E-05-3.04E-02
		Respiratory Disease	1.47E-04-1.23E-02
		DNA Replication, Recombination, and Repair	2.52E-04-2.44E-02
		Dermatological Diseases and Conditions	3.31E-04-2.31E-02

Supplementary Table 8. Pathway analysis of *ABHD8* overexpression models. Analyses were performed using Ingenuity Pathway Analysis, p-values from right-tailed Fisher's Exact Tests.

Analysis	Cell Line	Pathway	P-Value
Canonical Pathways	IOSE19	Hepatic Fibrosis / Hepatic Stellate Cell Activation	5.01E-12
		Caveolar-mediated Endocytosis Signaling	2.75E-10
		Granulocyte Adhesion and Diapedesis	1.17E-09
		Virus Entry via Endocytic Pathways	3.72E-07
		Role of Tissue Factor in Cancer	5.75E-07
		Agranulocyte Adhesion and Diapedesis	2.75E-06
		IL-8 Signaling	8.32E-06
		Communication between Innate and Adaptive Immune Cells	2.24E-05
		LPS/IL-1 Mediated Inhibition of RXR Function	6.92E-05
		Role of Macrophages, Fibroblasts and Endothelial Cells in Rheumatoid Arthritis	6.92E-05
	MCF10A	EIF2 Signaling	1.58E-14
		Oxidative Phosphorylation	2.19E-10
		Mitochondrial Dysfunction	7.94E-09
		Regulation of eIF4 and p70S6K Signaling	1.48E-06
		mTOR Signaling	6.31E-06
		Remodeling of Epithelial Adherens Junctions	1.20E-02
		Hepatic Fibrosis / Hepatic Stellate Cell Activation	1.23E-02
		Caveolar-mediated Endocytosis Signaling	1.35E-02
		Virus Entry via Endocytic Pathways	2.00E-02
		Glucocorticoid Receptor Signaling	2.63E-02
Disease and Functions	IOSE19	Cancer	7.83E-47-4.09E-08
		Cell Death and Survival	3.03E-41-2.22E-08
		Cellular Growth and Proliferation	3.59E-39-4.06E-08
		Cellular Movement	1.17E-38-3.43E-08
		Cardiovascular System Development and Function	6.97E-32-3.23E-08
		Organismal Survival	1.19E-29-2.67E-09
		Organismal Development	1.48E-27-3.28E-08
		Gastrointestinal Disease	3.16E-27-4.09E-08
		Embryonic Development	7.88E-26-2.1E-08
		Organismal Injury and Abnormalities	1.07E-24-3.95E-08
	MCF10A	Developmental Disorder	1.03E-08-1.22E-02
		Hereditary Disorder	1.03E-08-1.46E-02
		Metabolic Disease	1.03E-08-1.22E-02
		Neurological Disease	1.03E-08-1.22E-02

		Ophthalmic Disease	1.03E-08-3.52E-05
		Skeletal and Muscular Disorders	1.03E-08-1.22E-02
		Cancer	5.52E-08-1.49E-02
		Endocrine System Disorders	5.52E-08-1.46E-02
		Hematological Disease	2.48E-07-7.59E-03
		Cell-To-Cell Signaling and Interaction	6.71E-07-1.53E-02

Supplementary Table 9. Oligonucleotides used in this study.

3C NcoI primers	NcoI fragment (hg19 coordinates)	Sequence (5'to 3')
ABHD8 promoter bait	17,413,608	TCGTGACGGGCTTCTCTAAAGCTTTCAAGG
*Nco*I Fragment 1	17,413,229	CCTGGATTCTGGGGATCTGCTATACAAGTCC
*Nco*I Fragment 2	17,411,267	TCCTCAGCCAGCGCATAGAAGGTGTAGG
*Nco*I Fragment 3	17,408,710	GCTCAACTTGCCTGGCTGTTCAATCTTGC
*Nco*I Fragment 4	17,406,361	GGTTCATGTCCTCCCCCTACCCAGAAGG
*Nco*I Fragment 5	17,397,567	TGTCGCTCGCCTTCAGCCAGGAAGACAAGG
*Nco*I Fragment 6	17,396,773	CACCCACCTAGGGCTTCCACAATACACG
*Nco*I Fragment 7	17,395,991	CAGCTGAGCGCCTTCAGACTTTCATCC
*Nco*I Fragment 8	17,395,336	CTGTTCAGGCAGAGGTTACCCCAACAGG
*Nco*I Fragment 9	17,388,949	GGCCATGACTGGGACCTCTTCAGATCC
*Nco*I Fragment 10	17,384,724	GCTGGCTGAGGTTCAGATCTCAGATCTGC

Supplementary Table 10. PCR primers for luciferase assay constructs.

**Primer**	**Sequence (5' → 3')**
ABHD8_prom_For_XhoI	aaactcgagAACAGCCGGCCCTTTAAGTTCCTC
ABHD8_prom_Rev_NheI	aaagctagcGCAAGGAGCATTTAACCCCTCGTCC
PRE-A_For_BamHI	aaaggatccCACTAAAGCTCTCGAACCCCGATGTG
PRE-A_Rev_SalI	aaagtcgacACTGGAAGCCAGCAGCATTTGG
PRE-B_For_BamHI	aaaggatccCCATTTTGGCCAGGCTGTTCTCG
PRE-B_Rev_SalI	aaagtcgacCTGGACTGGTGGTTCTTAACTAAGGAGG

Supplementary References

1. Stevens, K.N. et al. 19p13.1 is a triple-negative-specific breast cancer susceptibility locus. Cancer Res 72, 1795-803 (2012). 2. Antoniou, A.C. et al. A locus on 19p13 modifies risk of breast cancer in BRCA1 mutation carriers and is associated with hormone receptor-negative breast cancer in the general population. Nat Genet 42, 885-92 (2010). 3. Bolton, K.L. et al. Common variants at 19p13 are associated with susceptibility to ovarian cancer. Nat Genet 42, 880-4 (2010).
